# Review: Clay-Modified Electrodes in Heterogeneous Electro-Fenton Process for Degradation of Organic Compounds: The Potential of Structural Fe(III) as Catalytic Sites

**DOI:** 10.3390/ma14247742

**Published:** 2021-12-15

**Authors:** Laura Cipriano Crapina, Liva Dzene, Jocelyne Brendlé, Florence Fourcade, Abdeltif Amrane, Lionel Limousy

**Affiliations:** 1 Institut de Science des Matériaux de Mulhouse CNRS UMR 7361, Université de Haute-Alsace, Université de Strasbourg, 3b Rue Alfred Werner, 68093 Mulhouse, France; laura.cipriano-crapina@uha.fr (L.C.C.); jocelyne.brendle@uha.fr (J.B.); lionel.limousy@uha.fr (L.L.); 2 Ecole Nationale Supérieure de Chimie de Rennes, Université Rennes, CNRS, UMR 6226, 35000 Rennes, France; florence.fourcade@univ-rennes1.fr (F.F.); abdeltif.amrane@univ-rennes1.fr (A.A.)

**Keywords:** Fenton, clay minerals, catalysis, environment, wastewater

## Abstract

Advanced oxidation processes are considered as a promising technology for the removal of persistent organic pollutants from industrial wastewaters. In particular, the heterogeneous electro-Fenton (HEF) process has several advantages such as allowing the working pH to be circumneutral or alkaline, recovering and reusing the catalyst and avoiding the release of iron in the environment as a secondary pollutant. Among different iron-containing catalysts, studies using clay-modified electrodes in HEF process are the focus in this review. Fe(III)/Fe(II) within the lattice of clay minerals can possibly serve as catalytic sites in HEF process. The description of the preparation and application of clay-modified electrodes in the degradation of model pollutants in HEF process is detailed in the review. The absence of mediators responsible for transferring electrons to structural Fe(III) and regenerating catalytic Fe(II) was considered as a milestone in the field. A comprehensive review of studies investigating the use of electron transfer mediators as well as the mechanism behind electron transfer from and to the clay mineral structure was assembled in order to uncover other milestones to be addressed in this study area.

## 1. Fenton Process for Wastewater Treatment

### 1.1. The Problematic Surrounding Industrial Wastewater Treatment

In Europe, around 54% of the water uptake for human activities is used in industry [1]. The industrial effluents are either treated in urban wastewater treatment plants (UWWTP) or in on-site independent wastewater treatment plants within the industrial facilities before being released into a water body. Treatment in the industrial facility is necessary for effluents containing pollutants that compromise operation of the UWWTP [1] or persist after this regular treatment. In the last decade, the prospect of environmental problems disclosed by researchers in the field and the rising awareness of the public about it has compelled governments to impose more restrict policies for management of industrial wastewater [2,3]. This context has increased research interest in new materials to overcome the limitations of available industrial wastewater treatments in order to support environmental policies and avoid criticism from state agencies and consumers.

### 1.2. Methods for Industrial Wastewater Treatment

The characteristics of industrial wastewater are often complex and particular to each manufacturing process and it may even vary when considering different days, weeks or sessions, depending on the company. The components of the matrix of wastewater define the most suitable line of treatment based on physicochemical treatments combined with a biological treatment [4]. The physicochemical treatments remove both insoluble and soluble pollutants [5,6]. The elimination of insoluble particles commonly involves treatments such as screening and sieving followed by the elimination of immiscible substances (oil and grease removal) [5]. As for soluble particles, physicochemical treatments are used to eliminate metals (e.g., chromium), anionic species (e.g., fluorides, phosphates and cyanide) and non-biodegradable organic compounds (e.g., phenolic compounds). Among physicochemical treatments, the most common treatments are based on stripping, absorption, ionic exchange and techniques using membranes, such as ultrafiltration [6]. In particular, the chemical treatment of wastewater with oxidizing or/and reducing agents can be used in order to convert soluble pollutants into insoluble compounds, which are later eliminated with another chemical treatment inducing precipitation of such compounds. The redox treatment can also be used to convert soluble pollutants into nontoxic compounds that may be later eliminated with biological treatment, for instance. In this case, another chemical treatment considered to be crucial to the following aerobic or anaerobic biological treatment is neutralization of wastewater that consists in adjusting the pH to circumneutral [3].

However, effluents commonly require a third treatment that involves the repetition of primary physicochemical treatments or secondary biological treatment in order to allow disposal of treated water following environmental policies or to have it reused in the original industrial process (recycled) and others such as fire and cooling systems [4]. Among chemical treatments, advanced oxidation processes (AOPs) have been studied to eliminate soluble organic compounds considered as persistent that are usually referred in the literature as persistent organic pollutants (POPs) [4,7,8]. In most of these processes, hydroxyl radicals (•OH) are used as the oxidizing agent that nonselectively degrade pollutants into water, carbon dioxide and inorganic salts [9]. However, the most cost-effective techniques to achieve mineralization of pollutants in wastewater involve using AOPs associated with electrochemical technology that are namely electrochemical advanced oxidation processes (EAOPs) as a pre- or post-treatment to biological treatment in order to convert pollutants into intermediate organic compounds [10]. The fundamental features of AOPs are recalled in the following topic with emphasis in Fenton-based processes and related technologies, such as the electro-Fenton process.

### 1.3. Heterogeneous Electro-Fenton Process

The acidification of effluents to pH 2–3 is required in the electro-Fenton process as in the conventional Fenton process mainly to avoid formation of iron sludge [2,8,11,12]. The use of heterogeneous catalysis in the electro-Fenton process can avoid acidification of effluents prior to the treatment that allows the working pH to be circumneutral or alkaline. The catalyst can be recovered and reused several times decreasing expenses related to reagents and avoiding the release of iron in the environment as a secondary pollutant [13]. The coupling of both heterogeneous catalysis and photo- or/and electro-chemistry have provided promising results and have been so far the most attractive version of Fenton process for full-scale treatment of industrial wastewater [14].

### 1.4. Materials in Heterogeneous Electro-Fenton Process

Early publications involving heterogeneous catalysis in the electro-Fenton process involved natural occurring iron minerals such as goethite, magnetite and pyrite [14]. However, iron oxides result in both homogeneous and heterogeneous catalysis depending on the pH [15,16,17], referred to here as pseudo-heterogeneous catalysts. Under acidic conditions, iron oxides tend to dissolve, releasing into solution Fe(II) or Fe(III) ions that are reduced to Fe(II) in the cathode (Equation (1)). The Fe(II) ions in solution follow then homogenous mechanism (Equation (2)) and Fe(II) or Fe(III) sites remaining in the surface participate in heterogeneous mechanism (Equation (3)). As the pH increases, the generation of •OH or HO_2_• radicals proceed mainly through surface-based mechanism upon structural ≡Fe(II)-OH (Equation (3)) and ≡Fe(III)-OH sites (Equation (4)), respectively [15]. However, the application of current across the system promotes partial reduction of structural ≡Fe(III)-OH to ≡Fe(II)-OH (Equation (5)) in minerals such as pyrite and iron oxides that culminate in dissolution and release of Fe(II) even under neutral conditions [14,18]. The electron transfer mechanism and reactions related to these minerals are discussed in detail elsewhere [14,16].

Fe(III) + e^−^ → Fe(II) (1)

Fe(II) + H_2_O_2_ → Fe(III) + •OH + OH^−^ (2)

≡Fe(II)−OH + H_2_O_2_ → ≡Fe(III)−OH + •OH + OH^−^ (3)

≡Fe(III)−OH + H_2_O_2_ → ≡Fe(II)−OH + HO_2_• + H^+^ (4)

≡Fe(III)−OH(H_2_O_2_)(ads) → ≡Fe(II)−OH(HO_2_•)(ads) + H^+^

≡Fe(II)−OH(HO_2_•)(ads) → ≡Fe(II)−OH + HO_2_•

≡Fe(III)−OH + e^−^ → ≡Fe(II)−OH (5)

In this context, iron-containing minerals may be particularly interesting for in situ remediation of sites in which the recuperation of catalyst and acidification of water or both are not feasible. For instance, natural pyrite was used for in situ remediation of groundwater. The dissolution of pyrite releases H^+^ until an equilibrium state that is proportional to initial concentration of this mineral. In this case, the acidification of groundwater coincided with self-dosage of Fe(II) catalyst providing optimal conditions for Fenton process [14,16].

Under other circumstances, clay minerals appear as a more stable heterogeneous catalyst because Fe(II) or Fe(III) sites are coordinated within the layers, where iron atoms tend to remain after reduction and reoxidation favoring the heterogeneous mechanism and long-term use of catalyst [18,19]. Besides, clay minerals are natural, abundant and inexpensive and their capacity to act simultaneous as an adsorbent and a catalyst for degradation of contaminants has further motivated studies about their application in the removal of organic and inorganic contaminants in soil and water [16,20,21].

The application of lamellar materials as a heterogeneous catalyst in the electro-Fenton process as well as the mechanism behind decomposition of H_2_O_2_ by structural Fe(III)/Fe(III) atoms occurring in the structure of natural and synthetic lamellar materials has not been exclusively reviewed before. This critical review aims to describe studies using clay minerals and clay-like materials as a heterogeneous catalyst in the electro-Fenton process. Further, it brings together studies investigating the contribution of iron atoms within the structure of clay minerals to the degradation of organic pollutants in wastewater and contaminated soils during the electro-Fenton process and related technologies.

## 2. Iron in Clay Mineral Structure and Its Reactivity

**Figure 1:**
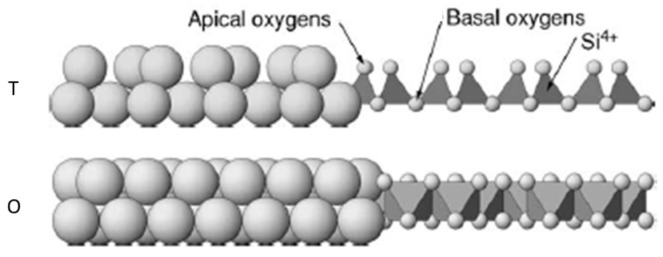
The tetrahedral (T) and octahedral (O) sheet represented with the sphere-packing (**left**) and polyhedral (**right**) model. Adapted from [21].

**Figure 2:**
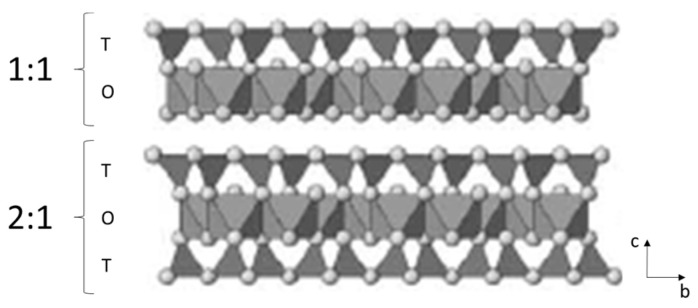
The representation of 1:1 (TO) and 2:1 (TOT) layer type with polyhedral model underlying the external surfaces of each type. Adapted from [21].

In soils, the mineral particles with size smaller than 2 µm are classified as clays. The particle size is used to separate clay from silt (2–50 µm) and sand (0.05–2.0 mm) [22]. The term “clay mineral” is used to describe phyllosilicates and other minerals, which provide plasticity to clay that harden upon drying or firing. Clay minerals may be of any crystallite size, but their unique properties are partly related to their small particle size and high surface area [23]. In addition, their physical and chemical properties depend upon their crystal structure being the basis for their classification. The basic structural units are tetrahedral (T) and octahedral (O) sheets (Figure 1) that stack to form layers and the number of sheets is used to divide the clay minerals into two major types: 1:1 (TO) and 2:1 (TOT) (Figure 2) [21]. For instance, Si(IV) are only able to fit into tetrahedral sites and Mg(II) as well as Fe(II) only to octahedral sites while Al(III) and Fe(III) have intermediate size being found coordinated in both tetrahedral and octahedral sites [21].

**Figure 3:**
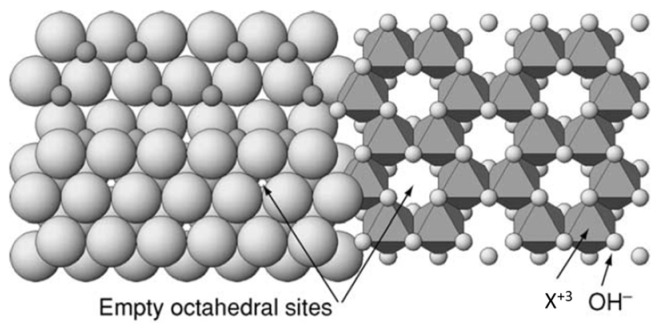
The representation of the octahedral sheet with dioctahedral arrangement in the sphere-packing (**left**) and polyhedral (**right**) model. Adapted from [21].

In the case of trivalent cations (e.g., Al(III) and Fe(III)), two out of three possible octahedral sites are occupied in order to maintain charge balance of the layered structure being this arrangement referred to as dioctahedral (Figure 3). As for the bivalent cations (e.g., Mg(II)), all possible octahedral sites are occupied being this arrangement referred to as trioctahedral [21].

**Figure 4:**
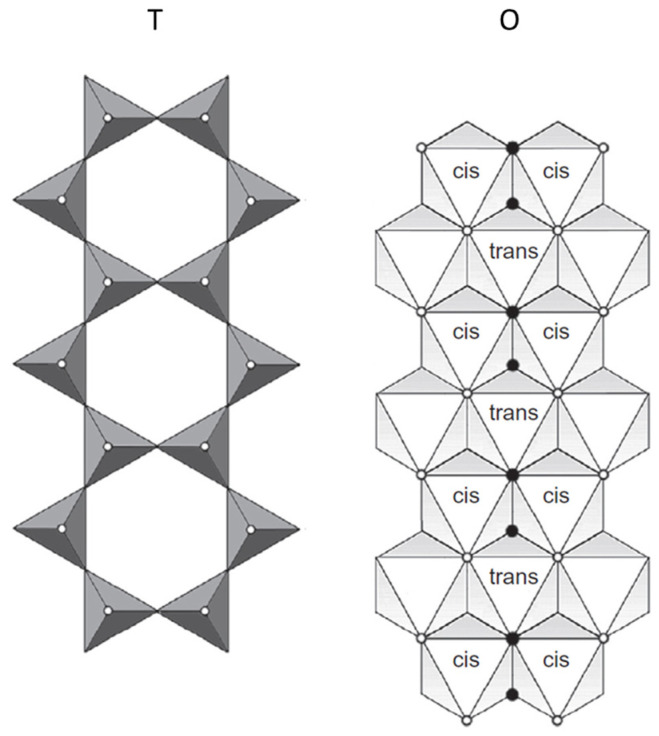
The structure of (T) tetrahedral sheet and octahedral sheet (O) in which the open circles represent the linkages between sheets. The octahedral sites are indicated as trans (M1) or cis (M2) based on the arrangement of hydroxyl ions. The filled circles represent the OH ions in trans-position, which introduce a plane of symmetry in the octahedral sheet. Reprinted from [22] with permission from Elsevier (License Number 5172351427456).

Besides, the octahedral sites in the dioctahedral sheet are discriminated depending on the arrangement of the hydroxyl ions bonded to the central site in trans or cis, being referred to as M1 or M2 sites, respectively [22]. The sites in which hydroxyl groups are located on opposite corners are considered trans-octahedral sites or M1 and those located on adjacent corners in the edge are cis or M2 (Figure 4) [24].

The 1:1 type clay minerals are based on one tetrahedral sheet sharing the apical oxygen (Figure 1) ions with an octahedral sheet (Figure 2 (TO)). In this group, the layers are neutral and adjacent layers are held together through hydrogen bonding between basal oxygen of the tetrahedral sheet and OH groups of the exterior plane of the octahedral sheet. The minerals representing this group are serpentine-kaolinite. Kaolinite represents the subgroup of 1:1 layer type minerals with a dioctahedral sheet and general chemical formula corresponding to Al_2_Si_2_O_5_(OH)_4_ [25]. The substitution of Al(III) by another metal cation is rare and the 1:1 layer shows little or no charge [21]. In the literature, the substitution of a metal cations by another with similar size is referred to as isomorphic substitution [26]. As for serpentine, it represents the subgroup with a trioctahedral sheet and general chemical formula corresponding to Mg_3_Si_2_O_5_(OH)_4_ [22].

The 2:1 layer structure is based on two tetrahedral sheets linked with an octahedral sheet (Figure 2 (TOT)). The minerals talc and pyrophyllite are 2:1 phyllosilicate minerals representing the group without any isomorphic substitution of cations in both tetrahedral and octahedral sheets. Pyrophyllite represents the subgroup with a dioctahedral sheet and general chemical formula corresponding to ^tet^Si_4_^oct^Al_2_O_10_(OH)_2_ while talc represents those with a trioctahedral sheet and general chemical formula corresponding to ^tet^Si_4_^oct^Mg_3_O_10_(OH)_2_ [21,25]. In this group, the electrically neutral layers interact with each other by weak Van der Walls forces [21].

The isomorphic substitution of atoms in the structure results in a charge deficit within the 2:1 layer that is compensated with interlayer cations, which are responsible for the cation exchange and swelling capacity of clay minerals [27]. If the layer charge is 1, the mineral is classified under the mica group. In this group, the occurrence of isomorphic substitution of Si(IV) in the tetrahedral sites by Al(III) results into a charge deficiency of 1. Muscovite represents the subgroup with a dioctahedral sheet and general chemical formula corresponding to ^int^K^tet^AlSi_3_^oct^Al_2_O_10_(OH)_2_ while phlogopite represents those with a trioctahedral sheet and general chemical formula corresponding to ^int^K^tet^AlSi_3_^oct^Mg_3_O_10_(OH)_2_ [21,28].

**Figure 5:**
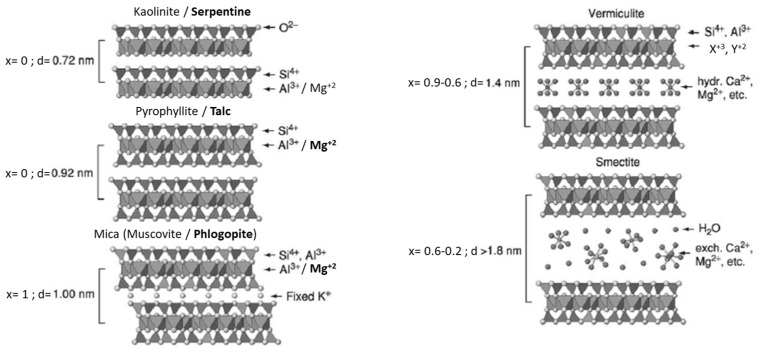
The representation of each group of phyllosilicates using the polyhedral model and respective charge (x) and layer to layer distance (d) Adapted from [21].

Besides, the occurrence of isomorphic substitutions in both tetrahedral and octahedral sheet results into a layer charge lower than 1. For instance, if the layer charge is between 0.6–0.9, the mineral is classified under the group of vermiculites. If layer charge is between 0.2–0.6, it is classified under the group of smectites [22]. The representation of each group is shown in Figure 5.

All phyllosilicates previously discussed exhibit a long-range order. However, other minerals such as allophane exhibits structural order only in the range of several nanometers showing an overall disordered structure. Allophane is an aluminosilicate mineral observed as spheres of 3.5–5.0 nm that are commonly associated with iron oxides and organic matter in volcanic ash soils. The spheres show variable amounts of O^2−^, OH^−^, Al(III) and Si(IV) that result in Si-O-Al bonds without an exact chemical formula or well-defined structure [21,29].

The majority of studies using clay minerals as a heterogeneous catalyst in the Fenton process involve natural or modified smectite minerals in which iron atoms occupy octahedral sites in the structure (Figure 4). The natural nontronites SWa-1, NAu-1 and NAu-2 as well as montmorillonites SWy-1 and SWy-2 are reference clay minerals that have been extensively evaluated and will be further discussed in this topic [30]. The organic matter appears strongly associated with the surface of allophane preventing purification of natural allophane even after treatment with hydrogen peroxide. For Garrido-Ramirez et al., the impossibility of purification exposed the prerequisite for the development of synthetic allophane to be used as heterogeneous catalyst in the electro-Fenton process [29,31].

### 2.1. Iron in Clay Mineral Structure

#### 2.1.1. Ferric Clay Minerals

Nontronite is classified as a 2:1 phyllosilicate under the group of smectites and subgroup of minerals with a dioctahedral sheet due to the predominant occupancy of octahedral sites by Fe(III) ions with minor isomorphic substitution by Al(III). The tetrahedral sites are predominantly occupied by Si(IV) with occasional substitution of Si(IV) by Al(III) or Fe(III) resulting in the general chemical formula: ^inter^M^+^_x+y_^tet^(Si_8−x−y_Al_x_Fe_y_^3+^)^Oct^(Fe^3+^_4−z_Al_z_)O_20_(OH)_4_·nH_2_O [24]. The naturally occurring nontronites Garfield, Panamint Valley, NG-1, SWa-1, NAu-1 and NAu-2 were used in studies later discussed in this review and then a brief description of each one is given in Table 1 regarding their origin, chemical formula and characteristics, such as layer charge, cation exchange capacity (CEC) and surface area.

Manceau, Lanson, et al. investigated in detail the crystal chemistry of Garfield, Panamint Valley, Swa-1 and NG-1 nontronites using several characterization techniques, such as chemical analysis, X-ray diffraction and X-ray absorption spectroscopy [24]. The authors concluded that the source of layer charge in these reference nontronite minerals raised from isomorphic substitutions, but the metal cations and their distribution between the tetrahedral and octahedral sheet varied among the samples. For Garfield, SWa-1 and NG-1, layer charge is mostly located in the tetrahedral sheet, while for Panamint Valley the charge located in the octahedral sheet is slightly higher than the one in tetrahedral sheet. Only NG-1 showed tetrahedral Fe(III), which corresponded to 14–20% of total iron in this reference sample. Besides, the authors reported that octahedral cations only occupied cis-coordinating octahedral sites (M2) in SWa-1 and further investigated the relative distribution of Fe, Al and Mg. The comparison of structural parameters obtained from modeling and reported data uncovered that iron atoms are not randomly distributed in the octahedral sites but clustered resulting in small magnetic domains, which are accountable for the magnetic properties observed for SWa-1.

Keeling reported the chemical composition of nontronites NAu-1 and NAu-2 [42]. The main chemical difference between these nontronites is that a higher aluminum content was reported for NAu-1 in comparison to NAu-2 in their respective general formulas: ^inter^M_+1.05_^tet^[Si_6.98_Al_1.02_]^oct^[Fe_3.68_Al_0.29_Mg_0.04_]O_20_(OH)_4_ and ^inter^M_+0.72_^tet^[Si_7.55_Al_0.45_]^oct^[Fe_3.83_Mg_0.05_]O_20_(OH)_4_. The NAu-1 was considered similar to previously reported Garfield nontonite. As for NAu-2, the authors stated insufficient Al(III) in the clay-size fraction and suggested the presence of Fe(III) in tetrahedral sites and Al(III) in the octahedral sites which explains the different chemical formula currently provided by the Clay Mineral Society [36] (Table 1). Therefore, the reference smectite minerals NG-1 and NAu-2 have structural Fe(III) located in both octahedral and tetrahedral sites but the tetrahedral Fe(III) is considered to occur in trace amounts [39] rendering controversial the role of tetrahedral Fe(III) in the reactivity observed for NAu-2 in the studies later discussed here [38,39]. Besides, Gorski, Aeschbacher, et al. reported that 2 wt% of total structural Fe(III) occupied tetrahedral sites in NAu-2 [30] while Joe-Wong, Brown, and Maher reported as much as 8 wt% in their study [43]. Besides, Gorski et al. reported that all structural Fe(III) in NAu-1 and mostly in NAu-2 were cis-coordinated in the octahedral sites [30]. For NAu-2, a second octahedral environment was observed and interpreted as trans or more accurately as a distorted cis-octahedral Fe(III) sites that were located near tetrahedral Fe(III) in the structure [30,39].

As for montmorillonite, it is also classified as 2:1 phyllosilicate under the group of smectites and subgroup of minerals with a dioctahedral sheet. However, the octahedral sites are predominantly occupied by Al(III) with only a small isomorphic substitution of Al(III) by Fe(III) while the tetrahedral sites are virtually occupied by Si(IV) resulting in the general chemical formula: ^inter^M_+x_^tet^(Si_8_)^oct^(Al,Fe_4−x_Mg_x_)O_20_(OH)_4_ [22]. For this reason, the layer charge of montmorillonite is primarily located in the octahedral sheet, but it is located in the tetrahedral sheet for nontronite [25]. The montmorillonites K10, STx-1, SWy-1 and SWy-2 were used in studies later discussed in this review and then a brief description of each one was displayed in Table 2. The montmorillonites SWy-1 and SWy-2 are reported under the same general chemical formula by the Clay Mineral Society [36] but different weight percentages of structural Fe(III) are reported in the literature. The SWy-1 showed 12.6 wt% of structural Fe (III) [44] while SWy-2 showed 2–2.3 wt% [19,41,45,46]. However, the structural Fe(III) is primarily located in cis-coordinating octahedral sites (M2) for both SWy-1 and SWy-2 [30,46]. As for STx-1, there is no relevant considerations about this montmorillonite in the literature aside from the relative lower structural Fe(III) content reported in the general formula [47].

#### 2.1.2. Reduction of Ferric Clay Minerals in Natural Systems

The subsurface soil-layers favor both oxidation states of structural iron. Firstly, the structural Fe(III) acts as an electron acceptor for microbial respiration in the absence of oxygen that is typical in this environment [51]. The microbial reduction of structural Fe(III) within smectites has been reviewed elsewhere [52]. Secondly, the subsurface-layer conditions favor electron transfer from contaminants to structural Fe(III) resulting simultaneously in structural Fe(II) and a radical cation, with the latter considered to be more toxic than the original contaminant [53]. However, this radical cation may polymerize with other radical cations contributing to decrease the environmental mobility of contaminants, such as phenol [54]. The resulting structural Fe(II) is reactive towards reduction of organic and inorganic contaminants in the surface of clay minerals and such reductive transformation of contaminants under anoxic conditions has been also reviewed elsewhere [55]. Besides, the clay minerals coexist with aqueous Fe(II) in slightly anoxic aquifers, river sediments and soils [37] and such Fe(II) ions may substitute exchangeable cations in the interlayer region as well as coordinate with the surface OH groups of clay minerals. The relative reactivity of those iron species was investigated on several publications that primarily intended to grasp the contribution of the subsurface soil-layers to the environmental fate of pollutants [55]. However, the studies investigating the relative reactivity of Fe(II) species under oxic conditions are rare in the literature. These studies are not only relevant to uncover environmental dynamics of pollutants in the surface-layers of soil but also the Fenton reactions during the heterogeneous electro-Fenton process. The few studies in this topic uncovered that •OH radicals are generated upon aeration of Fe(II)-containing clay minerals as shown in Equations (6)–(8) and mediate the oxidation of contaminants in the surface. Then, the transformation of contaminants in the surface of clay minerals is based on two different mechanisms depending on oxygenation of soils: direct and indirect mechanisms, which are favored under anoxic and oxic conditions, respectively [54,56,57]. The available literature investigating the reactivity of iron species located in the surface, interlayer or within the structure of clay minerals towards oxidation of contaminants, O_2_ and H_2_O_2_ will be discussed in order to highlight the contribution of each of these iron species to the generation of •OH radicals in the heterogeneous electro-Fenton process.

Fe(II) + O_2_ → Fe(III) + O_2_•^−^ (6)

Fe(II) +O_2_•^−^+ 2H^+^ → Fe(III) + H_2_O_2_ (7)

Fe(II) + H_2_O_2_ + H^+^ → Fe(III) + HO• + H_2_O (8)

### 2.2. Reactivity of Structural Iron

#### 2.2.1. The Relative Reactivity of Tetrahedral vs. Octahedral Fe(III)

Preliminary results by A. Neumann showed that tetrahedral Fe(III) undergoes reduction and reoxidation with redox active sites just like octahedral Fe(III) [58], which was investigated in detail in another study by this author dedicated to determine the reactivity of octahedral Fe(III) using a quantitative kinetic model [59]. Besides, A. Neumann showed that redox cycling of tetrahedral Fe(III) was possible for nontronites with low content of tetrahedral Fe(III) while investigating synthetic nontronites with different content of iron in the tetrahedral sheets [58]. The author argued that alterations in the clay mineral structure were responsible for compromising reversibility of electron transfer to and from tetrahedral Fe(III). Other studies that will be discussed in this topic extended this argument to tetrahedral and octahedral Fe(III) while discussing a decrease in reductive performance of iron-rich clay minerals after consecutive redox cycles.

Chen et al. investigated the generation of •OH radical from decomposition of H_2_O_2_ on the surface of four smectite minerals mediated by polyphenol molecules for degradation of diethyl phthalate under anoxic conditions [38]. The minerals showed different iron content and distribution within the layers. The SMF and FZ-10 were representative minerals for montmorillonite and NAu-1 and NAu-2 for nontronite. The authors observed that the concentration of •OH radicals correlated with the total iron content within the different minerals. The normalization of the time observed for initial formation of •OH radicals per total iron content revealed that structural Fe(III) of SMF, FZ-10 and Nau-1 showed similar activities while NAu-2 showed 15x higher activity than NAu-1. The superior performance observed for NAu-2 was attributed to the tetrahedral Fe(III) that corresponded to only 2 wt% of total iron content within NAu-2. A similar observation was reported by X. Liu et al. while investigating the generation of •OH radicals during oxygenation of reduced nontronite NAu-2 for oxidative attenuation of trichloroethylene at pH 7.5 [41]. In this study, the authors compared the yield of •OH radicals that was determined as the concentration of •OH radicals accumulated in the system upon oxygenation of 1 mM of structural Fe(II). The yield of •OH radicals observed for reduced NAu-2 was higher than the yield observed for reduced montmorillonite SWy-2. However, Z. Wang found similar performance for NAu-2 and montmorillonite K10 when comparing the rates associated to catalytic decomposition of H_2_O_2_ by NAu-1 and K10 [48]. The authors were investigating the mechanism behind the electron transfer from excited dye molecules to structural Fe(III) within both clay minerals and argued that only tetrahedral Fe(III) was redox active in NAu-2 because the iron content in the tetrahedral sheet of NAu-2 (2 wt%) was similar to the total iron content of K10 (2.05 wt%).

Nevertheless, Joe-Wong, Brown, and Maher emphasized that tetrahedral Fe(III) occurred in trace amounts within the structure of NAu-2 and argued that tetrahedral Fe(II) was not the main reductant of Cr(IV) ions sorbed in the edge of the layers under anoxic conditions [43]. At the same time, Zeng et al. argued that a fraction of •OH radicals generated in the surface of NAu-2 were depleted by structural Fe(II) [19]. The authors investigated the electron transfer from structural Fe(II) within reduced nontronite NAu-2 to O_2_ for generation of O_2_•^−^ and then •OH radicals for degradation of 1,4-dioxane at near neutral pH and absence of light. Their argument emerged from an experimental observation similar to X. Liu et al. [41] that NAu-2 displayed the highest iron content but the lowest accumulation of •OH radicals per mM of structural Fe(II) referred to as efficiency. The efficiency of •OH generation decreased as the concentration of NAu-2 increased and thus the concentration of structural Fe(II) which endorsed an inconsistence between the concentration of structural Fe(II) and generated •OH radicals. After three consecutive redox cycles, the efficiency of •OH generation for chemically reduced NAu-2 further decreased as a consequence of dehydroxylation reactions induced by consecutive reduction of structural Fe(III) to Fe(II) that resulted in the collapse of layers as shown by the decrease of specific surface area of cycled NAu-2. Similar observations were reported by Nzengung et al. [60]. The authors argued that inferior reactivity of reduced nontronite SWa-1 in comparison of reduced montmorillonite SWy-2 was a consequence of irreversible structural changes observed upon reduction of structural Fe(III) in smectite minerals with a higher Fe(III) content such as SWa-1 and NAu-2. In their study, the authors investigated the reductive dechlorination of perchloroethylene (PCE) at the surface of chemically reduced SWa-1 and SWy-2 under anoxic conditions. However, A. Neumann et al. reported that not only PCE but also trichloroethylene and chloroform were not susceptible to reductive transform by chemically reduced SWa-1 and thus such observation would rather be related to a secondary phase in the surface of SWa-1 and SWy-2 [61]. Indeed, Nzengung et al. [60] pointed out that SWa-1 and SWy-2 previously treated with sodium dithionite and washed before transformation experiments were unable to react with PCE. Besides, the authors pointed out that FeS2 was a possible secondary mineral phase in their system capable of slowly reacting with chlorinated organic compounds. This secondary phase was possibly responsible for the observation of a superior transformation of PCE in the presence of reduced SWa-1 and SWy-2 in comparison to a solution containing only dithionite. A similar observation was reported recently by Entwistle et al. [62]. The reductive transformation of PCE and trichloroethene (PCE) was observed in the presence of chemically reduced SWy-2 and 20 mM of Fe(II) ions under anoxic conditions due to the formation of a reactive mineral intermediates.

#### 2.2.2. Reactivity of Surface Adsorbed vs. Interlayer vs. Structural Fe(II)

Recently, Xie et al. investigated the role of Fe(II) species within different subsurface sediments in the generation of •OH radicals after 10 h of oxygenation for degradation of phenol in order to uncover the fate of contaminants in the subsurface-layers of soil perturbed with O_2_ [63]. In their study, the performance in phenol degradation, the concentration of •OH radicals and the extent of oxidation was evaluated for each of the Fe(II) species within the natural sediments that were original from beach sand, lakeshore and farmland soil. The concentration of •OH radicals was determined using an indirect assay based on the hydroxylation of benzoate into p-hydroxybenzoic acid by •OH. Under the same conditions used for phenol degradation with natural sediments, sodium benzoate was added instead of phenol and the resulting concentration of p-hydroxybenzoic acid was determinate and interpreted as the concentration of •OH. The accumulation of •OH radicals was closely related with removal of phenol by natural sediments that was negligible in beach sand, 41% in lakeshore and 52% in farmland sediments after 10h of oxygenation accomplished with exposition of the different sediments with phenol to air. However, beach sand and lakeshore sediments showed similar total iron content of 31.64 and 29.54 mg/g, respectively, while the farmland sediment showed higher iron content of 53.58 mg/g suggesting that total iron content was not the main parameter controlling generation of •OH radicals and thus degradation of phenol. From this total content, surface-adsorbed Fe(II) was minimal in beach sand, but 6.77 and 11.83 mg/g in lakeshore and farmland sediments, respectively. The Fe(II) as an exchangeable cation in the interlayer of clay minerals was 9.11, 9.81 and 19.05 mg/g in beach sand, lakeshore and farmland sediment, respectively. As for structural Fe(II), it was incorporated within carbonate, oxide and silicate minerals being 6.60, 4.27 and 23.29 mg/g the content associated within silicate minerals in beach sand, lakeshore and farmland sediments, respectively. Besides, the concentration of aqueous Fe(II) ions was below the detection limit in the suspension of all sediments. According to the authors, these observations showed that surface-adsorbed Fe(II) and structural Fe(II) were mainly responsible for firstly reducing O_2_ to H_2_O_2_ and secondly decomposing H_2_O_2_ in •OH radicals and thus degrading phenol. The authors confirmed this hypothesis evaluating the extent of oxidation for interlayer Fe(II) of clay minerals within beach sand sediment uncovering that as little as 3.6% oxidized after 10 h of oxygenation. The extent of oxidation of structural and surface adsorbed Fe(III) was also evaluated in the beach sand sediment as well as in other sediments. The fraction of oxidized surface-adsorbed Fe(II) in the total oxidized Fe(II) was 72.3% for lakeshore and 47.2% for farmland sediments. As for structural Fe(II), 21.4% and 49.7%, respectively. The differences in reactivity observed for Fe(II) species suggested that Fe(II) was adsorbed in different substrates and structural Fe(II) was coordinated within different minerals in the two sediments. Indeed, the clay mineral fraction in the farmland sediment corresponded to 13% and 51% in the lakeshore and farmland sediments, respectively.

Then, the authors developed a control experiment with different minerals in order to further elucidate the reactivity of Fe(II) species in the sediments towards oxidation of O_2_ and thus generation of •OH radicals for degradation of phenol. The minerals goethite, alumina, montmorillonite and kaolinite were evaluated as possible substrates to adsorption of Fe(II) in the sediments. The oxidation rate observed for Fe(II) adsorbed in the surface of each of the above minerals was much higher for Fe(II)/goethite and Fe(II)/alumina in comparison to Fe(II)/montmorillonite and Fe(II) kaolinite: Fe(II)/goethite > aqueous Fe(II) > Fe(II)/alumina >>> Fe(II)/montmorillonite and Fe(II) kaolinite. However, the accumulation of •OH radicals was much higher for Fe(II)/alumina than for Fe(II)/goethite that were similar to Fe(II)/kaolinite, but both were superior to Fe(II) montmorillonite: Fe(II)/alumina >>> Fe(II)/goethite~Fe(II) kaolinite >>> Fe(II) montmorillonite > aqueous Fe(II). Interestingly, the phenol degradation was higher for Fe(II)/kaolinite in comparison to Fe(II)/alumina and Fe(II) montmorillonite, but negligible for Fe(II)/goethite. The authors interpreted these observations assuming that adsorbed Fe(II) coordinated with surface groups of the different minerals using an inner-sphere mechanism in which the surface groups (≡X-O-) entered the coordination sphere of hexaquo Fe(II) ion through a ligand exchange process. The ≡X-O- ligands displayed different electron-donating abilities and as it increased so did the reactivity of surface-adsorbed Fe(II) species towards O_2_ as well as •OH radicals. In this case, the reactivity increased as ≡Si(IV)-O-Fe(II) < ≡Al(III)-O-Fe(II) < ≡Fe(III)-O-Fe(II) < ≡Fe(II)-O-Fe(II) < ≡HO-Fe(II) < ≡O--Fe(II). For instance, the highly reactive surface-adsorbed Fe(II) in goethite as ≡HO-Fe(II) probably interacted with O_2_ using an inner-sphere mechanism for electron transfer reaction which favored the generation of other reactive oxidants, such as Fe(IV) species, instead of •OH radicals, as previously investigated by [64]. The opposite argument is valid for poorly reactivate surface-adsorbed Fe(II) in alumina as ≡Al(III)-O-Fe(II) that interacted with O_2_ through full coordination environment using an outer-sphere mechanism for electron transfer reaction favoring the generation of •OH radicals. Previously, [65] uncovered that the electron transfer reaction between aqueous Fe(II) and molecular oxygen occurred in solution following the inner- or outer-sphere mechanism depending of the pH. The authors compared the electron transfer rate parameters calculated with a predictive model to experimental parameters available in the literature and concluded that the hydrolysis of hexaquo Fe(II) ions in basic pH favored the inner-sphere mechanism while fully coordinated Fe(II) ion in acid pH favored electron transfer to O_2_ using the outer-sphere mechanism. However, Xie et al. [63] complemented observations from Rosso and Morgan [65] as well as Hug and Leupin [64] proposing that the electron density of Fe(II) also influenced which mechanism the electron transfer reaction is compelled to proceed at near neutral pH. As for the relative performance of kaolinite and montmorillonite, Xie et al. predicted that the ligands ≡Al(III)O- and ≡Fe(III)O- should attribute greater reactivity to surface-adsorbed Fe(II) in montmorillonite than only ≡Al(III)O- in kaolinite. The authors interpreted their experimental observations for kaolinite and montmorillonite arguing that the interaction of surface-adsorbed Fe(II) with octahedral Fe(III) in montmorillonite stabilized Fe(II) against oxidation being responsible for the slower oxidation rates observed for farmland sediment [63].

Another control experiment evaluated the reactivity of structural Fe(II) in different coordination environments also reporting the oxidation rates, concentration of •OH radicals and degradation of phenol upon aeration of magnetite, montmorillonite, biotite or chlorite representing Fe(II)-bearing minerals. As previously mentioned, virtually all structural iron in montmorillonite is +3 and thus pretreatment of this clay mineral with an inorganic reductant (sodium dithionite) was required in order to reduce 42% of structural Fe(III) to Fe(II) in this experiment. Only the octahedral Fe(II) of reduced montmorillonite was greatly oxidized upon oxygenation resulting in the accumulation of •OH radicals and thus degradation of phenol. Magnetite, biotite and chlorite showed minimal oxidation of structural Fe(II) after 10 h of oxygenation. The authors discussed this experimental observation following the reasoning previously described but applied to Fe(II) coordinated with different ligands within the structure. The authors stated that the balance of electron poor (≡Si(IV)O-) and rich (≡HO-) ligands coordinated to structural Fe(II) in montmorillonite was responsible for superior performance of this mineral in comparison to iron oxide and other clay minerals [63]. This study was considered of great importance since it firstly introduced a parallel between the electron density of structural or surface-adsorbed Fe(II) and their reactivity towards oxidation and thus reduction of O_2_. The arguments proposed by the authors of this study enlightened the discussion of other studies investigating the mechanism behind the electron transfer to and from structural Fe(III) in Fe(III)-bearing clay mineral.

#### 2.2.3. The Secondary Role of Structural Fe(II) in the Generation of •OH Radicals

Gournis, Karakassides, and Petridis proposed that generation of hydroxyl radicals is primarily related to the availability of broken edge sites, which increase as the clay particle size decreases regardless of the iron configuration in the clay mineral structure [66]. The authors compared the accumulation of •OH radicals upon oxygenation of the suspension of synthetic hectorite mineral (laponite) (20 nm), montmorillonite SWy-1 (200 nm) and fluorohectorite (2000 nm) in which only montmorillonite exhibited structural Fe(III). The authors reported that accumulation of •OH radicals increased as clay particle size decreased as a consequence of the greater availability of broken edges in hectorite. In this context, the modulation of particle size for clay minerals exhibiting structural Fe(II) may be a promising topic for further investigations in the field.

## 3. Heterogeneous Catalyst Based on Clay Minerals for Heterogeneous Electro-Fenton Process

**Figure 6:**
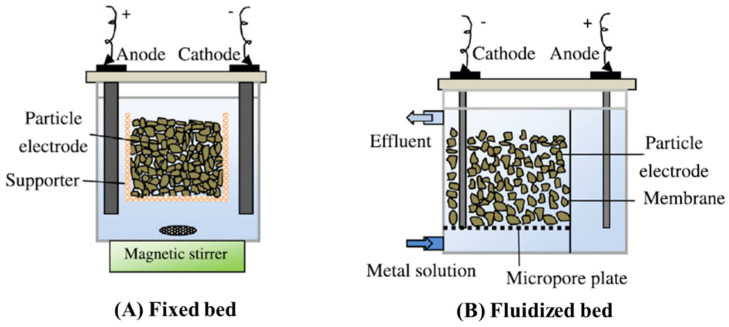
Scheme of three-dimensional electrochemical systems with heterogeneous catalyst restrained (**A**) or loosened (**B**) in solution as a bed in between the anode and the cathode (Reprinted from [68] with permission from Elsevier, License Number 5172360169697).

In the literature, the heterogeneous electro-Fenton process is mainly performed in two different arrangements in which the heterogeneous catalyst appears in solution or as a layer deposited upon the cathode. The most conventional configuration is based on the dispersion of heterogeneous catalyst in solution, usually referred to as a two-dimensional heterogeneous electro-Fenton system. The generation of •OH radicals mainly occurs in the liquid–solid interface following the heterogeneous mechanism, and mixing in this system is crucial to ensue effective mass transfer and then effective oxidation of pollutants [67]. Another configuration for the heterogeneous catalyst in solution is based on catalytic particles that are restrained (Figure 6A) or loosened (Figure 6B) as a bed in between the anode and the cathode in order to improve mass transfer and ionic charge distribution of conventional systems [68,69]. The resulting porosity of this configuration provides a higher specific surface area that not only shortens distance between reagents and electrode but also favors adsorption and electro-adsorption of pollutants resulting into superior percentages of removal. Further, more catalytic sites are available for organic compounds to undergo direct and/or indirect electro-oxidation depending on the material used for the fabrication of particles [68,70]. Many authors claim that the particles come to be polarized during electrolysis due to assemble of positive and negative ions from the electrolyte solution in their surface at opposite sides, which behave as an anode and cathode, respectively [67,68]. Then, each particle is presumed to act simultaneously as a heterogeneous catalyst and electrode being referred in the literature as catalytic particle electrode or simply particle electrode. For this reason, this configuration is commonly referred to as a three-dimensional heterogeneous electro-Fenton system, because the particles are considered as an extension of the electrode. However, the definition of electrode entails an electrical conductor/semiconductor [71] and the particles are not in contact with the current supply. Yet, the proximity between the particles and the electrode in the configuration shown in Figure 6B possibly promote the polarization of particles. At the same time, the electrical contact is not uniform and the current is not distributed homogeneously among the particles, which are the major drawbacks of this configuration [70]. In this context, the studies included in the following topic will consider particle electrodes essentially as particles which perform the role of heterogeneous catalyst arranged in different configurations in the HEF system.

### 3.1. Heterogeneous Catalyst as Particles

Among the studies using catalytic particles, only a few of them used particles based on lamellar materials, such as:

- Kaolin impregnated with ferric molybdate (Fe_2_(MoO_4_)_3_);
- Hematite (α-Fe_2_O_3_) and cuprous oxide (CuO) deposited on the surface of kaolin;
- Bentonite intercalated with iron oxide nanoparticles;
- Bentonite modified with different iron species: zero-valent iron (Fe^0^), magnetite (Fe_3_O_4_) or hematite (Fe_2_O_3_).

These few studies were reviewed here with particular focus on the preparation, characterization and optimization of the catalytic particles as well as their performance in the removal of pollutants and resistance to iron leaching as an indication of their performance upon reuse in several cycles.

**Figure 7:**
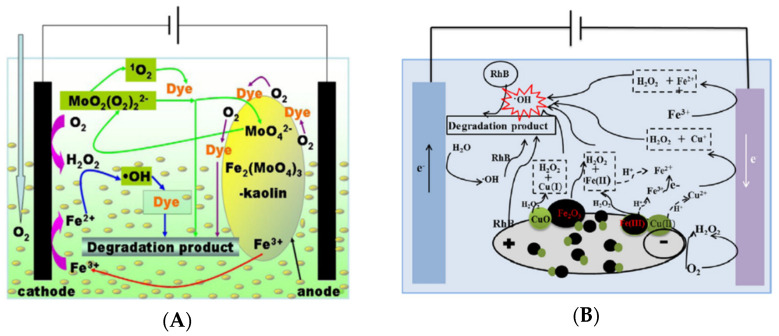
Scheme of three-dimensional electrochemical reactor using kaolin impregnated with (**A**) Fe_2_(MoO_4_)_3_ [69] (**B**) α-Fe_2_O_3_ and CuO [70] as a heterogeneous catalyst for removal of dye molecules along with the proposed pseudo-heterogeneous mechanism for degradation of pollutants. Reprinted from [69,70] with permission from Elsevier (License Numbers 5172360390748 and 5172360545687).

He et al. investigated kaolin impregnated with ferric molybdate, Fe_2_(MoO_4_)_3_, as catalytic particles in an undivided three-dimensional electrochemical reactor with graphite as the anode and cathode (6.5 cm × 4.5 cm) for degradation of methyl orange (MO) as shown in Figure 7A [69].

In their study, the authors also evaluated the performance of such a system with simulated azo dye wastewater containing not only MO, but also acid orange II, eriochrome blue R and methylene blue. In the electrochemical cell, the authors intended to generate •OH radicals with decomposition of H_2_O_2_ by Fe(III) as well as in the surface of the anode by anodic oxidation. Then, the system intended to associate heterogeneous electro-Fenton process based on electro-Fenton-like reactions and anodic oxidation for degradation of azo dyes. The particles were prepared upon mixing of Fe(III)-molybdate complex with kaolin for 2 h at room temperature following calcination at 450 °C. The physicochemical characterization of the Fe_2_(MoO_4_)_3_-modified kaolin (FM-kaolin-450) suggested homogeneous deposition of monoclinic Fe_2_(MoO_4_)_3_ on the surface of kaolin. First, authors discredited the removal of MO by adsorption showing that the use of FM-kaolin-450 alone was responsible for only 3% chemical oxygen demand (COD) removal after 60 min. Then, the authors investigated the influence of experimental parameters such as initial concentration of FM-kaolin-450 and MO, pH and current density in the COD removal. The catalytic particle under a concentration of 6.6 g/L in 0.05 M of Na_2_SO_4_ as a supporting electrolyte at pH 4.34 with aeration rate of 1 L/min and current intensity of 2.1 A achieved 92.48% COD removal after 60 min of electrolysis. Besides, degradation studies in the absence of FM-kaolin-450 showed that uncatalyzed H_2_O_2_ and anodic oxidation were responsible for 50.93% COD removal after 60 min confirming the essential role of •OH radicals from Fenton-like reactions on the particle for mineralization of MO. The same experimental conditions were used to degrade azo dyes in simulated wastewater and COD removal decreased as complexity of the molecular structure increased in particular for aromatic substituents being of 1.38% for eriochrome blue R, which showed two naphthalene substituents. Under optimal conditions, the reuse of FM-kaolin-450 in five consecutive runs was proposed to assess long-term stability of this catalytic particle. The COD removal decreased from 92.48% to 67.40% at the same time that concentration of Fe(II) in solution decreased from 0.36 to 0.1 mg/L in the electrochemical cell after five consecutive runs. The authors attributed loss of catalytic activity to adsorption of MO or by-products into active sites resulting in poisoning of the particle. However, the mechanism proposed for generation of •OH radicals involved Fe(II) previously converted in the cathode from Fe(III) supplied by FM-kaolin-450. In this system, the presence of Fe(II) in solution suggested that the process was rather classified as pseudo heterogeneous due to the considerable contribution of homogeneous catalysis.

More recently, kaolin was also used as a catalytic particle by B. Zhang et al. [70]. At this time, hematite (α-Fe_2_O_3_) and cuprous oxide (CuO) were deposited on the surface of kaolin particles for degradation of another dye, Rhodamine B (RhB) and evidence of heterogeneous catalysis was given in this study. Fe-Cu/kaolin was prepared with kaolin particles smaller than 149 µm mixed with Fe(II) and Cu(II) salts in aqueous solution for 1h. After, Fe-Cu modified kaolin was molded as a pellet of 3–5 mm of diameter, dried and calcined. The authors investigated the influence of parameters related to the preparation of Fe-Cu/kaolin in the removal performance of RhB. The authors concluded that the following parameters influenced the performance in the order: calcination temperature > ratio kaolin: pore-making agent > calcination time > amount of catalyst loading. The ideal conditions were the catalyst loading with 1% wt of Fe and Cu and calcination at 350 °C for 3 h. The physicochemical characterization of Fe-Cu/kaolin uncovered that spherical particles corresponding to iron oxide in the structural phase of hematite and cuprous oxide were deposited in the surface of kaolin particles resulting in an irregular surface that increased the specific surface area of kaolin particles. In the undivided electrochemical reactor, Fe-Cu/kaolin particles were loaded inside two baffles fixed between the graphite plates (6 cm × 4 cm) corresponding to the anode and cathode as shown in Figure 7B. First, the authors investigated operational parameters such as the initial concentration of particles, voltage, pH and aeration rate in order to maximize removal and degradation of RhB. The authors concluded that Fe-Cu/kaolin particle in a concentration of 30 g/L in 0.035 M of Na_2_SO_4_ at pH 6.7 with aeration rate of 0.8 L/min and under applied voltage of 10 V was able to promote removal of 94.8% and total organic carbon (TOC) removal of 58.1% for RhB after 60 min of electrolysis. However, the TOC removal was 58.1% while 14.4% for the system without any particle. Under the same conditions, the authors compared the performance of Fe-Cu/kaolin to other particles based on kaolin and activated carbon that were able to remove 80.3% and 75.2% of RhB, respectively. This observation indicated that adsorption and direct electrochemical oxidation reactions acted simultaneously with electro-Fenton-like reactions to discolor aqueous solution as anticipated by [72]. Upon reuse of Fe-Cu/kaolin particle, the performance of removal was reported to be slightly inferior after five consecutive runs, decreasing from 94.8% to approximately 82%. The authors attributed loss of catalytic activity to iron leaching from the particle into aqueous solution that corresponded to 0.08 mg/L at pH 6.7. Finally, the authors uncovered the contribution of •OH radical to removal of RhB using tert-butanol to trap •OH radicals. The results showed that removal of RhB decreased from 94.8% to 51.0% upon trapping of •OH radicals in the system, which confirmed the contribution of direct electrochemical reactions at the electrodes. Then, the authors proposed a possible mechanism for generation of •OH radical based on the decomposition of H_2_O_2_ with heterogeneous Fenton-like reactions. For heterogeneous reactions, the reduction of ≡Fe(III) to ≡Fe(II) by H_2_O_2_ adsorbed in the surface resulted in less reactive radical species HO_2_• and ≡Fe(II) that were able to further react with adsorbed H_2_O_2_ to generate •OH radical as in Equation (3). The contribution of Cu(II) was not only attributed to decomposition of H_2_O_2_ but also to the reaction of Cu(I) with Fe(III) resulting in Cu(II) and Fe(II) due to the difference in electrochemical potential of these atoms [70,73]. This mechanism was adapted from J. Wang et al. [73] that proposed this sequence of reactions while working with a bimetallic heterogeneous catalyst mainly composed by zero-valent iron and zero-valent copper, which are highly reactive in comparison to iron oxides [74]. Then, the role of Cu(II) as an electron transfer to Fe(III)/Fe(II) sites for reduction of Fe(III) to Fe(II) in the surface seems to be essential in this setting for successful heterogeneous generation of •OH radical in the particles. This conclusion was reinforced by the superior performance obtained with Fe-Cu/kaolin in comparison to Fe_2_(MoO_4_)_3_-modified kaolin particle, which were prepared only with Fe(III) by He et al. [69]. However, copper and cobalt are disclosed as contaminants in water bodies and leaching of these metals from the particles may be considered controversial when considering their application in wastewater treatment.

Another lamellar material that has been used as a catalytic particle is bentonite. (Nannan Qiao et al. investigated the use of bentonite intercalated with Fe*_x_*O*_y_* (Fe-Bent) as a catalytic particle for the degradation of the azo dye orange II [75]. First, the Fe*_x_*O*_y_* were obtained upon reaction of Fe(NO_3_)_3_ with extract of green tea and then loaded into bentonite upon mixing for 4 h. The Fe-Bent was dried at low pressure at 60 °C for 12 h and used as a catalytic particle in an electro-Fenton system. The physicochemical characterization of Fe-Bent showed that iron oxide nanoparticles associated with plant-based polyphenol were accommodated in the interlayer space of bentonite. In the undivided electrochemical reactor, the Fe-Bent were packed in between the graphite plates (3.5 cm × 6 cm) corresponding to the cathode and anode being only 1 cm from each other for degradation of orange II under a current density of 14.29 mA/cm^2^.

The authors investigated operational parameters such as pH, initial concentration of Fe-Bent, electrolyte and current efficiency in order to maximize the performance of Fe-Bent as catalytic particle. The ideal conditions resulted in 98.9% of discoloration and 71.6% of COD removal for the dye orange II in aqueous solution with 0.05 M of Na_2_SO_4_ as an electrolyte, 0.5 g of Fe-Bent and pH 6.4 after 60 min of electrolysis. Under these conditions, the authors disclosed that 65.8% of color removal resulted from adsorption of orange II in the particle, which was acting simultaneously as an adsorbent and a catalyst. The intensity of current efficient (ICE) was higher in the presence than in the absence of particles indicating superior performance of proposed configuration in the generation of •OH radicals and thus in the degradation of orange II. The color removal decreased from 98.9% to 94.3% after the reuse of Fe-Bent in five consecutive cycles in this system, but respective COD removals were not reported at this time. The authors proposed that such decrease in catalytic activity was due to the formation of iron oxyhydroxides on the surface of the particle or leaching of Fe(II) in solution. Then, the authors monitored the concentration of Fe(II) during electrolysis showing that the concentration of Fe(II) in solution was approximately 8.5 mg/L after 5 min decreasing to 3.84 mg/L after 60 min. This result illustrates consumption of Fe(II) for generation of •OH radical and ineffective regeneration in the cathode possibly due to their precipitation as iron oxyhydroxides (pH 6.4) suggesting a significant contribution of homogeneous catalysis for generation of •OH radicals in this system. Indeed, the authors proposed a possible mechanism for degradation of orange II with •OH radical generated by homogeneous catalysis. It has to be noted that the color removal was 55.0% in the absence of Fe-Bent indicating the contribution of direct electrochemical reaction of orange II in the electrode in this system.

The same group of researchers published another study investigating bentonite modified with different iron species: zero-valent iron (Fe^0^), magnetite (Fe_3_O_4_) or hematite (Fe_2_O_3_) as a catalytic particle for degradation of phenol in an heterogeneous electro-Fenton system [76]. The physicochemical characterization of the different Fe-Bent showed that iron species Fe^0^ and Fe_3_O_4_ disrupted the layered structure while Fe_2_O_3_ maintained the regular stacking of bentonite. In the undivided electrochemical reactor, 2 g of the different Fe-Bent were packed in between the graphite plates (3.5 cm × 6 cm) corresponding to the cathode and anode for degradation of phenol under pH 6.4 and current density of 125 mA/cm^2^. Under these conditions, the authors reported the highest color removal when using Fe^0^-Bent after 180 min of electrolysis. The authors investigated the concentration of Fe(II) during electrolysis and uncovered higher iron leaching for Fe^0^-Bent than from Fe_2_O_3_-Bent or even Fe_3_O_4_-Bent. The authors concluded that the superior performance of Fe^0^-Bent was due to Fe(III) in solution leached from these particles. Upon the iron leaching profile, the authors concluded that a decrease of iron leaching from Fe^0^-Bent with reaction time indicated heterogeneous catalysis and adsorption of iron ions on the particle. As previously mentioned, the decrease observed in the concentration of Fe(II) in solution during electrolysis could be also interpreted as the consumption of Fe(II) and low regeneration of Fe(II) at the cathode due to complexation of Fe(III) with by-products from the degradation of phenol or/and their precipitation as oxyhydroxides. In this study, the authors used salicylic acid to determine the presence of •OH radical and uncovered a higher amount of •OH radicals for Fe_2_O_3_-Bent and Fe_3_O_4_-Bent in comparison to Fe^0^-Bent. For oxides, an abrupt increase in the concentration of •OH radical was initially observed followed by a dramatic decrease during electrolysis. The authors remarked that the presence of higher •OH radical was not related to higher removal of phenol but favored scavenger of •OH by H_2_O_2_ in the system. These results uncovered that Fe_2_O_3_-Bent and Fe_3_O_4_-Bent allowed homogeneous and heterogeneous generation of •OH radical, but were less effective in the removal and degradation of phenol under applied experimental conditions that favored side reactions and limited the performance of those catalytic particles.

### 3.2. Heterogeneous Catalyst upon Cathode

The use of cathodic materials as a primary or secondary solid support for iron species is considered as an ongoing trend in the electro-Fenton process [13]. The coating of the cathode with a solid catalyst combines convenience of heterogeneous process, such as extended range of working pH and reuse of catalyst with possible enhancement in the electroactivity of the cathode and then in the performance of electro-mediated oxidation of pollutants in the heterogeneous electro-Fenton systems. Most of the studies are based on coating the cathode with a secondary iron-supporting material such as iron minerals (pyrite [77], magnetite [78,79,80] and hematite [15,81,82]), zero-valent iron nanoparticles [74], chitosan [83] or zeolite [84] impregnated with iron oxide. More recently, cathodic materials with a three-dimensional porous structure able to support iron oxides in their composition referred to as Fe-carbon aerogel achieved remarkable performance in the degradation of pollutants under circumneutral conditions, but their multistep preparation involving complex techniques, such as supercritical drying, still prevents their practical application as a heterogeneous catalyst in the electro-Fenton process [14,15].

Only few studies have been dedicated to evaluating the modification of electrodes with iron-rich lamellar materials. Previously, clay-modified electrodes were described in the literature for fabrication of sensors and biosensors for detection of H_2_O_2_ and glucose, for example [85,86,87]. However, the use of clay minerals as an electrode coating for the electro-Fenton process has not yet been explored to our knowledge. The studies discussed in the previous topic did not explore the possibility of recovering the cathode with clay minerals probably due to limitation regarding their physical-chemical properties. Only few studies have been dedicated to clay-like materials as a solid iron support used to coat the cathode in the heterogeneous electro-Fenton process that were found to be: layered double hydroxides, allophane and hybrid organic-inorganic talc-like materials.

#### 3.2.1. Layered Double Hydroxides

**Figure 8:**
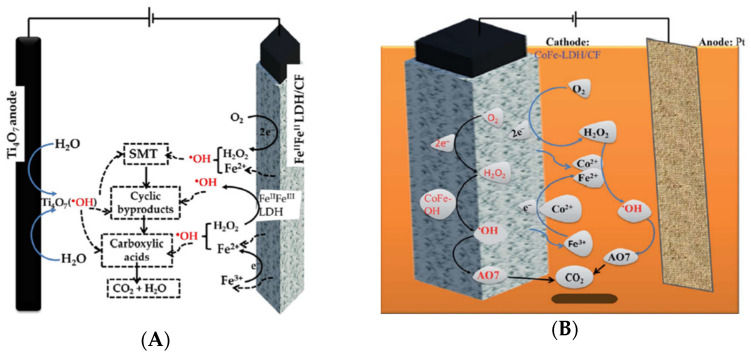
Scheme of two-dimensional electrochemical reactor using carbon felt coated with layered double hydroxides (LDH) with (**A**) Fe(III) and Fe(II) incorporated in the lattice for degradation of sulfamethoxazole [88] or (**B**) Cu(II) and Fe(III) incorporated in the lattice for degradation of orange II [89] along with respective mechanism proposed for degradation of pollutants. Reprinted from [88] with permission from Elsevier (License Number 5172361206614) and [89] with permission from Royal Society of Chemistry (Order License ID 1155310-1).

Ganiyu et al. used a carbon felt (CF) electrode coated with layered double hydroxides (LDH) modified with Fe(III) and Fe(II) as the cathode in an electrochemical cell for degradation of the antibiotic sulfamethoxazole using the heterogeneous electro-Fenton process [88]. The composite cathode Fe(III)Fe(II)-LDH/CF was prepared using an in situ solvothermal process based on hydrothermal treatment of an aqueous solution containing Fe(II)/Fe(III) salts in the presence of CF, which was previously cleaned with HNO_3_ using urea and ammonium fluoride as precipitation agents. The physicochemical characterization of Fe(III)Fe(II)-LDH/CF uncovered that both Fe(III) and Fe(II) atoms were incorporated in the lattice of LDH deposited upon CF fibers as particles resulting in an irregular surface with a porous structure. The Fe(III)Fe(II)-LDH layered structure was highly oriented and well-crystalized in the presence and absence of CF. However, a side phase of iron oxides in the form of hematite and maghemite was also observed on the surface of CF fibers. The modified Fe(III)Fe(II)-LDH/CF electrode showed higher electrical conductivity and potential of electron transfer than CF alone thus improving cathodic generation of H_2_O_2_ and regeneration of Fe(II). In the undivided electrochemical cell, Fe(III)/Fe(II)-LDH/CF (4.5 cm × 1 cm × 1.27 cm) was associated with a thin film of sub-stoichiometric titanium oxide (Ti_4_O_7_) (24 cm^2^) as the anode submerged in 0.05 M Na_2_SO_4_ electrolyte for degradation of 0.2 mM of antibiotic under applied current density of 7.5 mA/cm^2^ and aeration rate of 1 L/min for 8 h (Figure 8A). In these conditions, the authors investigated the contribution of •OH radical generated via water oxidation on the surface of Ti_4_O_7_ anode to performance of the system using only CF that resulted in TOC removal of 64%, 63% and 53% in pH 3, 6 and 9, respectively. With Fe(III)Fe(II)-LDH/CF, the TOC removal increased to 97%, 93% and 90% at pH 3, 6 and 9, respectively, while only 1% of TOC removal was observed for LDH/CF cathode showing that removal of antibiotic by adsorption contributed minimally for observed results. Then, the performance of Fe(III)/Fe(II)-LDH/CF was compared to the homogeneous electro-Fenton process using CF cathode and 0.2 mM of Fe(II) ions that resulted in TOC removal of 77%, 69% and 58% in pH 3, 6 and 9, respectively, being inferior to percentages achieved with Fe(III)Fe(II)-LDH/CF as the working electrode. Upon increase of the pH, concentration of total iron ions in solution decreased from approximately 9, 1 and 0.5 mg/L in pH 3, 6 and 9, respectively. Upon this result, authors proposed that mechanism for generation of •OH radical by the Fenton reaction mainly occurred on the surface because less than 10% of the TOC removal were dependent on the pH. At pH 3, the Fe(III) and Fe(II) ions released from the Fe(II)Fe(III)-LDH/CF contributed to the generation of •OH radical and slightly increased the percentage of TOC removal. Fe(III)/Fe(II) ions in solution could also result from dissolution of iron oxide side phases. As the pH increased to 6 or 9 in solution, the heterogeneous catalysis came to be even more dominant than homogeneous because LDH is nearly insoluble under basic conditions. This conclusion corroborates the observed increase in electrolysis time for complete degradation of antibiotic of 40, 120 and 240 min for pH 3, 6 and 9, respectively. As previously mentioned, there are additional steps in heterogeneous catalysis such as diffusion and adsorption of antibiotic on active oxidant sites on the surface of the cathode. As for the mechanism of heterogeneous catalysis, the authors adapted the mechanism described for iron oxides assuming that Fe(II)/Fe(III) occupying the crystallographic sites in LDH would be similar. The authors justified the choice by the fact that the reaction mechanism and kinetics behind the decomposition of H_2_O_2_ by structural metal atoms in lamellar materials is not well-established in the literature. Indeed, few studies are available and the effort of researchers in this topic will be discussed later in this review. At last, the performance of Fe(II)Fe(III)-LDH/CF cathode was investigated after reuse for 10 consecutive runs of 4 h each instead of 8 h at pH 6. After 10 runs, TOC removal decreased less than 17% and electrolysis time for complete degradation increased from 1 h to 2 h as a consequence of mechanical wearing since Fe(II)Fe(III)-LDH was stable at pH 6. Most importantly, the authors showed that this composite cathode is a promising pretreatment for toxic effluents evaluating survival of bacteria *Vibrio fischeri* with the Microtox^®^ method. In this system, 40 and 70 min of electrolysis was required to convert sulfamethoxazole into biodegradable by products in pH 3 and 6, respectively.

The same group of researchers used also mixed Cu(II)/Fe(III) system instead of Fe(II)/Fe(III) to prepare LDH coating CF that was used as a cathode in an electro-Fenton system for degradation of the azo dye Acid Orange II (Figure 8B) [89]. The association of iron to other transition metals in solution or inside the heterogeneous catalyst was previously attempted by several publications that concluded it may induce reduction of Fe(III) to Fe(II) and as a consequence enhance the performance of heterogeneous catalyst [14,90,91,92]. As previously mentioned, copper and cobalt are disclosed as contaminants in water bodies and thus the authors considered leaching from the electrode to be detrimental to the system [88].

More recently, the performance of a heterogeneous electro-Fenton (HEF) system using the Fe(II)/Fe(III)-LDH/CF cathode as a post-treatment of concentrate landfill leachates was investigated by El Kateb et al. [93]. The conventional treatment of landfill leachates involves processing in a membrane bioreactor (MBR) followed by nanofiltration in order to concentrate biorefractory compounds in the residue. In this study, the authors intended to treat this concentrate residue with previously described Fe(II)/Fe(III)-LDH/CF cathode in order to remove biorefractory compounds and thus reduce toxicity of residue before landfill discharge or recirculation in the MBR enhancing the cost-effectiveness of this process. However, at this time, the Fe(II)/Fe(III)-LDH/CF cathode (20 cm × 6 cm) was placed on the inner wall of the cylindrical reactor while rectangular titanium substrate coated with a thin film of Ti_4_O_7_ (32 cm^2^) were placed in the center. The electrochemical cell was filled with 220 mL of concentrated residue and electrolysis was carried out without initial pH adjustment and in the absence of supporting electrolyte (Na_2_SO_4_) under an applied current density of 4.2 mA/cm^2^ for 8 h. In these conditions, dissolved organic carbon (DOC) removal of 77% was observed but 16 ± 2% of this value were attributed to volatilization of organic compounds upon bubbling of oxygen and adsorption of hydrophobic organic compounds into CF cathode. The authors observed that initial pH decrease to 4–3 during electrolysis due to buildup of short-chain carboxylic acids resulting from oxidation of more complex organic compounds in the residue. This observation corroborates with the minor but progressive loss of catalytic activity observed upon the reuse of Fe(II)/Fe(III)-LDH/CF in four consecutive cycles that was attributed to iron leaching and mechanical wearing of Fe(II)/Fe(III)-LDH from CF and implied the necessity of regular pH control in order to prevent depletion of catalyst. Upon increase of applied current density to 8.3 mA/cm^2^, the DOC removal increased to 96% and the energy consumption of the system increased too. Then, the authors proposed to recirculate the concentrated residue treated in the HEF system in the MBR in order to enhance cost effectiveness of the process. At last, the authors investigated the unfolding of organic and inorganic compounds in the system during electrolysis. For that, the organic matter was investigated by three-dimensional fluorescence spectroscopy, low molecular weight carboxylic acids were identified and quantified with ion-exclusion high-pressure liquid chromatography (HPLC) and inorganic ions NO_3_^−^, NH_4_^+^, ClO_3_^−^ and ClO_4_^−^ were quantified with ion chromatography. After, the authors concluded that biological post-treatment with MBR would decrease electrolysis time to 4 h and increase overall performance to complete COD removal, but greater retention of proteins in pretreatment and biological nitrification of NH_4_^+^ in post-treatment would be demanded from MBR. The authors were able to carefully investigate the combination of electrochemical advanced oxidation processes (EAOPs) using a lamellar composite cathode and biological treatment to manage highly complex effluents and thus provided valuable knowledge towards the implementation of this approach in treatment of real effluents.

#### 3.2.2. Allophane

The catalytic activity of materials generally increases as size decreases because more atoms are accommodated in the surface-liquid interface and as a consequence available to act as active sites upon adsorption of organic compounds. Aside from size, the morphology, structure and composition of core and surface are parameters influencing activity of particle catalysts with less than 100 nm of diameter [16]. The deposition of nanoparticles in the surface of cathodic materials is an alternative to avoid loss of catalytic activity due to conglomeration and loss of nanoparticles in the supernatant while isolating and rinsing for reuse which also avoid the release of this material in the environmental [16].

Garrido-Ramírez et al. used allophane with iron atoms covalently bonded to the surface as Si-O-Fe and Al-O-Fe instead of deposited as iron oxides or oxyhydroxides to coat a glassy carbon disk electrode [31]. This cathode was then used in an electrochemical cell for degradation of the herbicide atrazine. The Fe-allophane was prepared using coprecipitation of silica and aluminum salts to obtain a final ratio SiO_2_/Al_2_O_3_ of approximately 2.2 and then 2 g were impregnated with 6 wt% of Fe(III) in aqueous solution upon evaporation of water as previously described by the same group of researchers [29]. The physicochemical characterization uncovered Fe-allophane with a hydrous feldspathoid-like structure. Then, Fe-allophane was mixed with graphite and a commercial perfluorinated ion-exchange resin (Nafion^®^) to prepare a disc-shaped glassy carbon electrodes (GCE) (0.5 cm^2^). The catalytic activity of Fe-allophane/GCE was investigated in an undivided electrochemical cell equipped with the Fe-allophane/GCE as the cathode, a platinum wire (data not shown) as anode and silver/silver chloride as the reference electrode. All electrodes were submerged in a solution with 90% 0.1 M of Na_2_SO_4_ and 10% of methanol in order to increase solubility of 46 mM of atrazine under saturation of oxygen and applied potential of −1.04 V. First, the influence of the weight ratio Fe-allophane: graphite and pH in the catalytic activity of electrode was investigated in pH 3 after 8 h of electrolysis. The authors evaluated the rate decay of atrazine and concluded that *w/w* ratio 0.6 was ideal in comparison to 0.2 and 1. As for the pH, the authors reported that the decay of atrazine was approximately twice faster and resulted in decay of 100% in the concentration of atrazine in pH 3 in comparison to 89% in pH 4 and 76% in pH 6. These results corroborate with observed increasing concentration of Fe(III) ions in solution at pH 3, 4 and 6 suggesting that homogeneous and heterogeneous catalysis were responsible for decay of atrazine in the system. At last, the authors compared atrazine rate decay in the heterogeneous electro-Fenton process using Fe-allophane/GCE to the heterogeneous Fenton process using only Fe-allophane, which was four times slower in pH 3. The authors attributed this result to the continuous generation of H_2_O_2_ and regeneration of Fe(II) in the cathode. This result could also be attributed to prior conversion of leached Fe(III) ions to Fe(II) able to decompose H_2_O_2_ into •OH radicals.

Later, the authors further refined Fe-allophane/GCE cathode with inclusion of Cu(II) resulting in a bimetallic Fe-Cu-allophane/GCE [94] in which Fe(III) atoms were bonded to the surface through Si-O-Fe or Si-O-Cu bonds [29]. At this time, the 1 g of allophane was impregnated with different weight percentages of iron and then of copper in aqueous solution upon evaporation of water. The data of physicochemical analysis corroborates with previous study, but the authors claimed the presence of very small particles of iron, copper or bimetallic oxides on the surface even in the absence of reflections related to iron or copper oxides in their X-ray diffraction pattern [29]. The same undivided electrochemical cell was used but with the Fe-Cu-allophane/GCE as the cathode submerged in a solution with 0.05 M of Na_2_SO_4_ and 0.5 mM of phenol under saturation of oxygen and applied potential of −0.6 V. First, the influence of Fe(III) and Cu(II) content in COD removal was investigated using Fe_3+x_Cu_3-x_-allophane/CGE in pH 5.5 after 4 h of electrolysis. The percentage of COD removal for phenol was of 48%, 48%, 68% and 69% for Fe, Fe_3_Cu_3_, Fe_4.5_Cu_1.5_ and Fe_5.4_Cu_0.6_-allophane/GCE, respectively. The authors concluded that the Cu(II) ions act in synergy with iron in the generation of •OH radical only if incorporated at small portions being able to significantly increase catalytic activity. These results corroborate with the concentration of Fe(III) and Cu(II) ions observed in solution suggesting homogeneous catalysis contributed for decay of phenol in this system. The authors proposed a mechanism including the synergetic action of Fe(III) and Cu(II) in the surface and solution for generation of •OH radicals in the system. For them, the presence of Cu(II) in the surface of Fe-allophane increased the number of active sites for activation of H_2_O_2_. Furthermore, Cu(II) ions reacted with Fe(III) to accelerate regeneration of Fe(II) ions responsible for the decomposition of H_2_O_2_ to •OH radicals [88]. The leaching of copper is considered detrimental due to toxicity of this transition metal. In this context, further improvement in the stability of such modified electrode is recommended in order to benefit from Fe(III) and Cu(II) synergetic action in the generation of •OH radicals.

In another study, the Fe-allophane was investigated upon a Ti/TiO_2_ cathode by the same group of researchers for degradation of the dye methylene blue under acidic conditions by a spectro-electrochemical process [95]. The irradiation of TiO_2_ with UV light photogenerates an electron-hole pair: the electron is able to react with O_2_ and the hole with adsorbed H_2_O to generate hydroperoxyl (HO_2_•) and hydroxyl (•OH) radicals, respectively. In this case, the layer of Fe-allophane aimed to extend absorption of solar radiation (photocatalysis) as well as to increase surface area and adsorption of pollutants. For this purpose, a titanium plate was modified with TiO_2_ using an electrochemical anodizing method and then coated with Fe-allophane using a previously described method for glassy carbon electrodes (GCE) [29,31]. The physicochemical characterization of Ti/TiO_2_/Fe-allophane electrode showed an increase in specific surface area due to the formation of additional meso and micropores. First, the novel electrode was evaluated by cyclic voltammetry using an undivided electrochemical cell equipped with Ti/TiO_2_/Fe-allophane as the cathode (6 cm × 2.5 cm with 0.25 mm of thickness), platinum wire (data not shown) as the anode and a saturated calomel electrode as the reference electrode. All electrodes were submerged in a nitrogen-saturated solution with 0.1 M of Na_2_SO_4_ as the supporting electrolyte and pH 3. The voltammetric response observed for Ti/TiO_2_/Fe-allophane electrode corresponded to a quasireversible process for Ti(III)/Ti(IV) couple while a reversible process was observed for Ti/TiO_2_ electrode. The authors argued that Fe-allophane layer comprised the diffusion through the film and consequently altered the response of the electrode. The degradation studies were performed under an electrochemical cell with similar configuration. At this time, the electrodes were submerged in a solution 0.1 M of Na_2_SO_4_ at pH 3 with 15 mg/L of methylene blue under applied potential of −1.0 V for 3 h in the absence or presence of UV light. The authors focused only on the generation of oxygen reactive species by Ti(III)/Ti(IV) pair for degradation of methylene blue. First, the contribution of adsorption was investigated and 79.7% of color removal in the system was related to adsorption of positive methylene blue molecules into the negative surface of Ti/TiO_2_/Fe-allophane in pH 3. The electrochemical process achieved 80.1% of color removal that increased to 86.9% in the presence of UV-light (photo-electrochemical process). Thus, the authors concluded that Fe-allophane removed dye from solution by adsorption behaving as a “concentrator” matrix instead of a heterogeneous catalyst. The data related to the concentration of iron ions in solution and reuse of Ti/TiO_2_/Fe-allophane were not given, preventing the assessment of the potential of Ti/TiO_2_/Fe-allophane to mediate indirect oxidation of methylene blue with the Fenton process.

#### 3.2.3. Clay-like Hybrid Inorganic-Organic Materials

The organic compounds near or adsorbed on the surface of the cathode are readily accessible to react with heterogeneously generated •OH radicals [96]. In this context, the adjustment of surface properties of clay minerals is a new promising trend in the heterogeneous electro-Fenton process. The anchoring of amino chains in the interlayer space was able to enhance water solubility of iron-rich claylike material [97,98]. Lee, Kim, et al. explored the capacity of structural Fe(III) to decompose H_2_O_2_ in order to act as a colorimetric sensor in immunoassays [97]. The peroxidase-like mimetic activity of Fe-aminoclay was confirmed in circumneutral pH and later further explored for the Fenton process in another study [98]. The Fe-aminoclay was exfoliated in water to fabricate a self-assembled system with water-soluble graphene oxide that was capable of acting as a heterogeneous catalyst for Fenton process resulting in complete discoloration of an aqueous solutions of anionic and cationic organic dyes at pH 6 after 5 h.

This study encouraged the use of iron-rich organic-inorganic lamellar materials to modify cathodic materials for the electro-Fenton process. Miron et al. coated carbon felt (CF) with a talc-like-hybrid (TLH) containing Fe(III) in the octahedral sheet to be used as the cathode in an electrochemical cell for degradation of a tri-iodide compound used as a contrasting agent in X-ray imaging with the heterogeneous electro-Fenton process [99]. The Fe-aminoclay was prepared based on a sol-gel method previously described by Lee, Kim, et al. [97] that involved addition of amino-propyltriethoxysilane (APTES) into a solution of FeCl3 in ethanol. First, the authors evaluated the aspect of Fe-aminoclay layer upon the carbon felt fibers when using different weight ratios of Fe(III) and Mg(II) in the condensation of a solid phase in the absence or presence of carbon felt. The physical-chemical characterization of Fe(III)-TLH/CF uncovered that Fe(III)-TLH was deposited upon carbon felt fiber as a thin, continuous and homogeneous film only upon addition of APTES in the presence of carbon felt. This aspect was only observed in the presence of 100 wt% of Fe(III) that was incorporated to the octahedral sheets of TLH and possibly also as a side phase of iron oxides in the surface of CF fibers. The modified 100% Fe(III)-TLH/CF electrode showed higher electron transfer resistance than CF alone, which possibly comprised generation of H_2_O_2_ and regeneration of Fe(II). In this study, the undivided electrochemical cell was equipped with Fe(III)-TLH/CF (2 cm × 2 cm and thickness of 0.6 cm) as the cathode, a platinum plate (32 cm^2^) as the anode and a saturated calomel electrode (SCE) as the reference electrode. All electrodes were submerged in an electrolyte solution with 0.05 M of Na_2_SO_4_ at pH 3 saturated with O_2_ for degradation of the tri-iodide compound under applied potential of −0.5 V/SCE for 4 h. In these conditions, the use of Fe(III)-TLH/CF as the cathode was able to increase performance of carbon felt from 70.1% to 98% in the degradation of the tri-iodide compound. When using only carbon felt, no ferrous ions were added to the electrochemical cell. The performance of carbon felt cathode was attributed to the direct electrochemical oxidation of the tri-iodide compound in the cathode and by H_2_O_2_. The increase in performance with Fe(III)-TLH/CF was attributed to indirect oxidation with •OH radical generated with catalytic decomposition of H_2_O_2_ by Fe(II), which resulted from the reduction of leached Fe(III) ions from Fe(III)-TLH/CF in the cathode. However, further studies are needed to assess contribution of adsorption and leaching of Fe(III) from the Fe(III)-TLH/CF to confirm the heterogeneous mechanism of catalysis and reusability. Furthermore, the evaluation of Fe(III)-TLH/CF performance in pH circumneutral would support practical application of this composite electrode.

## 4. Use of Electrochemistry for Reduction of Fe(III) to Fe(II) in Clay Mineral Structure

Recently, the mechanism behind generation of •OH radical through decomposition of H_2_O_2_ in the solid–liquid interface of iron-supporting catalysts was summarized in a review article [14]. Another review article detailed the mechanism behind the decomposition of H_2_O_2_ on the surface of iron oxides [96]. However, these publications did not report the mechanisms involving clay minerals. Despite having some similarities, the exposed crystallographic surfaces of clay minerals are expected to differ from iron oxides, and as a consequence their reactivity towards H_2_O_2_. In this context, this topic aims to discuss the publications investigating the mechanism behind decomposition of H_2_O_2_ by structural Fe(III)/Fe(II) in clay minerals.

The oxidation state +III is observed for the great majority of iron atoms existing in the lattice of 2:1 phyllosilicates, such as nontronite [24,100]. The decomposition of H_2_O_2_ is not observed in the presence of structural Fe(III) [101,102]. This observation possibly encouraged the use of interlayer or surface iron oxides as a catalyst site instead of structural Fe(III) within lamellar materials. A common ground was found around the statement that partial reduction of structural Fe(III) to Fe(II) is essential for the performance of iron-containing lamellar materials as a heterogeneous catalyst [37,103,104]. Indeed, the presence of structural Fe(II) smectite clay minerals increased the rate and extent in which organic compounds were degraded in anoxic [105] and oxic conditions [102].

Thus, the reduction of Fe(III) to Fe(II) appears to be the rate-limiting step of the whole heterogeneous electro-Fenton process [14]. In the literature, the reduction of structural Fe(III) to Fe(II) was accomplished using inorganic reducing agents, such as sodium dithionite [106,107], microorganisms, such as bacteria [108,109] as well as the irradiation of UV-vis light and electrochemistry in the presence of compounds acting as electron transfer mediators [101,110]. The reactions are often related to color change of clay minerals from yellow to green or blue-green [111,112].

### 4.1. The Essential Role of Electron Transfer Mediators as Activators of Clay-Modified Electrodes

The application of a current across the electrochemical cell promotes partial reduction of structural Fe(III) to Fe(II) within iron oxides and the extent of reduction increases in the presence of zero-valent iron and other transition metals [14]. However, the reduction of structural Fe(III) within lamellar material is more complex [51] and direct electron-transfer from the electrode surface to fixed structural Fe(III) and Fe(II) sites within the lattice is not observed in natural iron-rich clay minerals [110,113,114].

The use of electroactive species to reduce structural Fe(III) to Fe(II) and thus activate clay-modified electrodes was first described by Oyama and Anson [110]. In their study, the clay-modified electrode was based on a graphite electrode coated with sodium montmorillonite. However, the clay-modified electrode was only active in the presence of [Ru(NH_3_)_6_]^+3^ ions in solution being able then to electroreduce H_2_O_2_ to H_2_O. The authors confirmed that the [Ru(NH_3_)_6_]^+3^ ions were not able to catalyze reduction of H_2_O_2_ neither in solution or in the presence of graphite electrode confirming the essential role of clay coating for the activity of the electrode. At the same time, the authors did not observe a voltammetry wave for coated graphite electrodes in the absence or presence of H_2_O_2_ suggesting that [Ru(NH_3_)_6_]^+3/+2^ ions mediated electron transfer from the electrode surface to structural Fe(III) sites. The evidence of such electron transfer was seen in the enhancement of the cathodic peak current observed in the first cyclic voltammetric scan of iron-smectite clay modified electrode with [Ru(NH_3_)_6_]^+3^ ions incorporated in the cation exchangeable sites. The authors proposed that behind such enhancement of the cathodic peak current was the electrochemically reduced [Ru(NH_3_)_6_]^+2^ (Equation (9)) subsequently inducing the reduction of structural Fe(III) in the lattice of montmorillonite (Equation (10)). The [Ru(NH_3_)_6_]^+3^ ions do not react with structural Fe(II) and thus anodic peak current remained virtually identical. In the following scans, the cathodic peak current declined. Then, the exposure of the once cycled cathode to H_2_O_2_ which react with structural Fe(II) resulted in H_2_O (Equation (11)) and structural Fe(III) nearly restored enhancement observed during the first scan. This study was considered of great importance because it proved experimentally the role of structural Fe(II) not only as a catalyst for activation of H_2_O_2_ but also as a sink of electro-generated H_2_O_2_.

[Ru(NH_3_)_6_]^+3^ + e^−^ → [Ru(NH_3_)_6_]^+2^ (9)

[Ru(NH_3_)_6_]^+2^ + structFe(III) → [[Ru(NH_3_)_6_]^+3^ + structFe(II) (10)

H_2_O_2_ + 2H^+^ + 2structFe(II) → 2H_2_O + 2structFe(III) (11)

Other electroactive compounds such as ML_3_^+2^, in which M = Fe, Ru, Os and L = 2,2′bipyridine (bpy), 1,10-phenanthroline (phen) as well as methyviologen (MV^+2^), were investigated with regards to the electrochemical activity of montmorillonite coated electrode in other studies [115,116,117,118,119].

King, Nocera, and Pinnavaia investigated graphite electrode coated with a film of montmorillonite clay that was pre-exchanged with electro-active ML_3_^+2^ cations in order to uncover to which extent the cation exchangeable sites contributed to the electroactivity of clay-modified electrodes [117]. However, under these conditions, all cations were inactive. For instance, [Os(bpy)_3_]^+2^ substituting Na^+^ in the cation exchange sites in different percentages were used to coat a graphite electrode. This [Os(bpy)_3_]^+2^-montmorillonite/graphite electrode showed no response during cyclic voltammetry studies. However, the studies performed with [Os(bpy)_3_]^+2^ in the electrolyte and 80%-pre-exchanged-[Os(bpy)_3_]^+2^-montimorillonite/graphite electrode resulted in waves related to [Os(bpy)_3_]^+2/+3^ redox couple. A similar profile was observed upon use of Na-montmorillonite/graphite electrode with [Os(bpy)_3_]^+2^ in the electrolyte. The authors interpreted this observation as an evidence that cations in excess of the cation exchange capacity were responsible to reduce structural Fe(III) to Fe(II) (Equation (12)). After, the use of different particle sizes to coat the graphite electrode uncovered the contribution of edge surfaces. The smaller particles have greater edge surface but similar basal surface. The graphite electrode coated with clay particles with smaller size, such as hectorite (<50 nm) showed greater current response in comparison to montmorillonite (<200 nm) and fluorohectorite (<1000 nm) modified electrodes suggesting that electrochemical activity was related to edge instead of basal surfaces. At last, the authors concluded that the cations in excess of the cation exchange capacity bounded at edge surface sites were responsible for ion pairing mechanism that activated their clay-modified electrode.

[Os(bpy)_3_]^+2^ + structFe(III) → [Os(bpy)_3_]^+3^ + structFe(II) (12)

Rudzinski and Bard also investigated the electroactive compounds [Ru(NH_3_)_6_]^+2^ and [Ru(NH_3_)_6_]^+3^ incorporated in Texas montmorillonite (STx-1) deposited as a thin film in a In_2_O_3_ conducting glass electrode [116]. The authors considered the contribution of structural Fe(III) in the cyclic voltammetry responses of the STx-1/In_2_O electrode and attributed the wave at +0.6 V to reduction of structural Fe(III) by [Ru(NH_3_)_6_]^+2^ (Equation (10)). In a later study, Villemure and Bard observed that MV^+2^ pre-exchanged montmorillonite electrodes prepared as previously described were electro-inactive [118]. This observation corroborates with conclusions of King, Nocera, and Pinnavaia that the addition of electron transfer mediators in the electrolyte is crucial for the success of clay-modified electrodes [117].

In particular, Oyama and Anson encourage further investigation on the use of other clay-modified electrodes as well as on the role of structural iron towards electroactivity of such composite materials. At the end of their study [110], the authors anticipated that iron-rich smectite clay, such as nontronite, would possibly result in superior electro-reduction of H_2_O_2_. Later, Zen, Jeng, and Chen based their study on this premise and investigated the performance of glassy carbon electrodes coated with nontronite for electroreduction of H_2_O_2_ using MV^+2^ as an electron transfer mediator [113]. Here, the clay referred to as nontronite is ferruginous smectite SWa-1. The form Na^+^-SWa-1 was deposited as a film on the surface of glassy carbon electrodes (GCE). The authors used [Ru(NH_3_)_6_]^+3^ and montmorillonite SWy-1 to prepare electrodes under the same protocol in order to compare results with previous studies. The GCE and then Na^+^-SWa-1/GCE were evaluated with cyclic voltammetry in phosphate buffer as the electrolyte at pH 8. After addition of only H_2_O_2_ to the electrolyte solution, no wave was observed for both electrodes. However, after addition of H_2_O_2_ and MV^+2^, the wave observed for reduction of H_2_O_2_ with GCE was significantly enhanced with Na^+^-SWa-1/GCE. The performance of Na^+^-SWa-1/GCE was compared to Na^+^-SWy-1/GCE with and without H_2_O_2_ in the electrolyte solution containing MV^+2^. In the cyclic voltammogram without H_2_O_2_, the reduction peak of MV^+2^ is higher for Na^+^-SWa-1/GCE than for Na^+^-SWy-1/GCE uncovering that the amount of MV^+2^ electroactive is higher for Na^+^-SWa-1/GCE as a consequence of higher number of iron sites for SWa-1. In the cyclic voltammogram with H_2_O_2_, a major change in the wave profile was observed and the catalytic limiting current remains higher for Na^+^-SWa-1/GCE than for Na^+^-SWy-1/GCE. Then, the authors investigated the influence of pH in order to confirm the contribution of edge faces to the electroactivity of MV^+2^ towards Na^+^-SWa-1/GCE. As the pH decreased from 8 to 2.9, the electroactivity was significantly reduced uncovering that protonation of the structure decreased concentration of active species adsorbed on the surface. However, the authors interpreted this and previous observations as an evidence of the contribution of tetrahedral iron sites to superior electroactivity of Na^+^-SWa-1/GCE. However, Manceau, Lanson, et al. performed a detailed investigation of the crystal chemistry of several reference nontronite samples including SWa-1 and reported the absence of tetrahedral occupancy for this nontronite [24], which invalidates a major contribution of tetrahedral sites to the redox activity of clay-modified electrodes. As for the mechanism, Zen, Jeng, and Chen proposed that MV^+2^ reduced to MV^+^ on the electrode (Equation (13)) is able to transfer an electron from the electrode surface to the substrate to reduce structural Fe(III) to Fe(II) and regenerate MV^+2^ (Equation (14)) and then structural Fe(II) is responsible for the reduction H_2_O_2_ to H_2_O (Equation (9)).

MV^+2^ + e^−^ → MV^+^ (13)

MV^+^ + structFe(III) → MV^+2^ + structFe(II) (14)

Later, Hu investigated the electroreduction of O_2_ using a carbon paste electrode (CPE) modified with montmorillonite (SWy-2) using methylviologen (MV^+2^) as an electron transfer mediator [47]. The carbon paste was mixed with Na^+^-SWy-2 and MV^+2^ and after solvent evaporation this mixture was pressed into a hollow electrode body. The performance of MV^+2^-SWy-2/CP electrode was evaluated with cyclic voltammetry in an air-saturated phosphate buffer solution at pH 7 under applied potential of 0.1 V and −0.9 V/SCE. The reduction peak reduced significantly in a deoxygenated buffer solution indicating an on-going catalytic reduction of O_2_. The authors pointed out that the MV^+2^-SWy-2 restrained MV^+2^ to the electrode because release of MV^+2^ was observed for CPE/ MV^+2^. Under acidic conditions the number of MV^+2^ bounded to the surface decreased resulting in the absence of a reduction peak at pH 4.4. Besides pH, the authors also investigated the influence of the amount of Na^+^-SWy-2 uncovering that two reduction peaks were observed when lower amounts were used to prepare the MV^+2^-SWy-2/CPE. The first event was reported as the reduction of O_2_ using two electrons to H_2_O_2_ and the second event as further reduction of H_2_O_2_ using two electrons to H_2_O and such profile was first reported in their study. As for the mechanism, the authors proposed that the first event proceeds with electrochemically generated MV^+^ (Equation (13)) alone reducing O_2_ to O_2_^−^ (Equation (15)) which further reacted with H^+^ to result in O_2_ and H_2_O_2_ (equation (16)). Then, as previously mentioned, the structural Fe(II) generated upon oxidation of MV^+^ (Equation (14)) reduced H_2_O_2_ to H_2_O (Equation (9)). In this case, the essential role of structural Fe(III)/Fe(II) was confirmed employing kaolin (KGa-2) instead of MV^+2^-SWy-2 for preparation of a MV^+2^-KGa-2/CPE that showed a small peak current in comparison to MV^+2^-SWy-2/CPE. However, the authors did not discuss the role of MV^+2^-SWy-2 in the first catalytic event considering that the electrode CPE/MV^+2^ showed no current response to oxygen reduction in the voltammogram. This study was also considered of great importance because it sustained the electrogeneration of H_2_O_2_ by electroactive species incorporated to the electrode that are essential to the generation of Fe(II), which are the active catalytic sites. Thus, the incorporation of electron transfer mediators to clay-modified electrode are promising approach to improve the performance of iron-rich lamellar materials as heterogeneous catalyst in EF process.

MV^+^ + O_2_ → MV^+2^ + O_2_^−^ (15)

2O_2_^−^ + 2H^+^ → O_2_ + H_2_O_2_ (16)

The previous studies showed the activation of structural Fe(III) using different electron transfer mediators adsorbed at the edge sites. However, Xiang and Villemure investigated synthetic Fe(II)-smectite clay prepared under reductive conditions and deposited upon an In-doped SnO_2_ conductive support (SnO_2_:In) as a thin film with approximately 300 nm of thickness [114]. In this study, the authors observed for the first time the oxidation of Fe(II) to Fe(III) in the absence of electron transfer mediator compounds under anoxic conditions. Under the same conditions, the natural ferruginous smectite SWa-1/SnO_2_:In showed to be inactive. The incorporation of Ni(II) in the lattice of the iron-smectite clay enhanced the peak current, which was six times greater upon incorporation of 14% of Ni(II) while nickel-smectite clay/SnO_2_:In were inactive. However, the authors concluded that only the structural Fe(II) existing in the lattice due to reductive conditions used during synthesis accounted for the voltammetric wave observed in the above conditions. For scanning potential of 0.65 V to 0.2 V, no voltammetric wave was seen suggesting inactivity of structural Fe(III) sites towards reduction to Fe(II). In the second scanning of 0.65 V to 1.1 V, no voltammetric wave was seen suggesting that all electroactive structural Fe(II) sites oxidized to Fe(III) during the first scan. In regard of the possible performance of synthetic Fe(II)-smectite clay as a heterogeneous catalyst, it would be restricted to single use due to the electro-inactivity of resulting Fe(III). This limitation was overcome in the presence of a mixture of electroactive species [Ru(NH_3_)_6_]^+3^ and [Fe(bpy)_3_]^+2^ which were able to not only mediate oxidation of a greater fraction of structural Fe(II) with reduction of [Fe(bpy)_3_]^+3^ to [Fe(bpy)_3_]^+2^ (Equation (17)), but also to regenerate electroactive Fe(II) sites within the lattice upon reduction of structural Fe(III) with [Ru(NH_3_)_6_]^+2^ to [Ru(NH_3_)_6_]^+3^ (Equations (9) and (10)). In the context of HEF process, the redox cycling of Fe(III)/Fe(II) is fundamental not only to the performance, but to the feasibility of this material in practical applications, because it supports the reuse of catalyst over several cycles.

[Fe(bpy)_3_]^+3^ + structFe(II) → [Fe(bpy)_3_]^+2^ + structFe(III) (17)

### 4.2. The Mechanism behind the Electron Transfer to and from Structural Fe(III)/Fe(II)

Previously, studies focusing on the electrochemical activation of structural iron within clay minerals were outlined while the effort of authors to propose a mechanism was highlighted in order to be further discussed in this topic. The reduction of structural Fe(III) involves more than just an electron transfer to octahedral sites in the lattice, because structural rearrangements are demanded in order to maintain charge balance and accommodate atomic size of oxidation state +II [30,105]. Studies investigating structural changes resulting from reduction, oxidation and re-reduction in order to propose a mechanism of electron transfer to and from structural Fe(II)/(III) will be reported in this topic. The mechanism behind the reduction of structural Fe(III) to Fe(II) was investigated in several studies but outstanding contribution was achieved in the following studies [24,34,59,112,120,121,122].

J. W. Stucki and Roth used electron spectroscopy for chemical analysis (ESCA) currently known as X-ray photoelectron spectroscopy (XPS) and Mössbauer Spectroscopy [112]. A Garfield nontronite saturated with Na^+^ was treated with reducing agent sodium dithionite or hydrazine in order to convert structural Fe(III) into Fe(II) and reoxidation was achieved by exposing films to O_2_ and water vapor or submerging in O_2_ bubbling solution, respectively. The authors observed that the mechanism behind the reduction of structural Fe(III) depended of the amount of iron sites in the octahedral sheet. If the iron content was below 53 mmol/100 g, the coordination environment of iron sites in the octahedral sheet remained unaltered and an increase of the net negative charge was observed for the layer. However, if the iron content was above 53 mmol/100 g, the authors observed that the excess of negative charge was eliminated throughout changes in the structure. The initial argument of coordination of iron contracting from six- to fivefold coordination was discredited with studies using 3H as a label element [120] and modeling based on experimental data published for iron-rich smectites [34,121]. In particular, the exhaustive characterization of the crystallochemical structure of reduced Garfield nontronite by Manceau, Drits, et al. was key to build a model uncovering that iron atoms migrated from cis-sites to vacant adjacent trans-sites in the octahedral sheet during reduction process [121]. This migration preserved the sixfold coordination and created trioctahedral domains in the layer. Besides, basal oxygen atoms altered their orientation in the surface resulting in a flat basal surface, which is typical of trioctahedral layer silicates. The migration from cis to trans-sites of structural Fe(II) atoms resulted in a dehydroxylation reaction of protonated OH groups previously coordinated with Fe(III). It then resulted in vacancies in O_2_^−^ atoms, which were bordering the structure. The charge of O_2_^−^ forces the closest iron atom towards the edge of clusters and further incorporation of protons from solution to compensate their charge. The protonation of structural OH and incorporation of cations into the interlayer region were observed following the alteration of the oxidation state of iron atoms in the structure in order to compensate net negative charge accumulating in the layer. The model proposed by Manceau, Lanson, et al. also supported the conclusion that the distribution of Fe(III)/(II), Al(III) and Mg(II) in the SWa-1 was not random, but favored the presence of Fe(III)/(II) domains segregated from each other by Al(III), Mg(II) and empty octahedral sites [24]. The displacement of structural Fe(II) atoms towards the edge site upon redox cycling may account for the conclusion of studies cited in the previous topic that established that electron-transfer occur in the edge-sites of clay minerals. This sequence of publications regarding the reduction of structural Fe(III) to Fe(II) in dioctahedral smectites was assembled as a review article [105].

More recently, Yuan et al. used several spectroscopic characterization techniques to assess the structural rearrangements resulting from aeration of a suspension of reduced NAu-2 uncovering that the oxidation of structural Fe(II) proceeds as a two-stage process [123]. The authors uncovered that different rates of oxidation are observed depending on the location of Fe(II) in the lattice (edge or interior) as well as on the coordination environment of Fe(II) in di or tri-octahedral sites. From the Mössbauer spectra, the authors determined that the majority of Fe(II) within reduced NAu-2 occupied trans-coordinated octahedral sites which corroborates with structural rearrangements previously proposed in which the Fe(III) migrates from cis to adjacent vacant trans-coordinated octahedral sites upon reduction of Fe(III) to Fe(II). However, two different trans-coordinated octahedral sites were identified in the spectra. The regular trans-coordinated octahedral sites were attributed to di-octahedral domains as Al(III)-Fe(II) and Fe(II)-Fe(III) while distorted trans-coordinated octahedral sites were attributed to tri-octahedral domains Fe(II)-Fe(II)-Fe(II) being referred to as Fe(II)-1 and Fe(II)-2, respectively. Upon aeration, the disappearance of Fe(II)-2 from the Mössbauer spectra within 15 min indicated that tri-octahedral domains containing Fe(II)-Fe(II)-Fe(II) and edge Fe(II) were quickly oxidized while the resulting dioctahedral domains Fe(II)-Fe(II) and others such as Fe(II)-Al(III) and Fe(II)-Fe(III) were slowly oxidized in the interior of the structure.

The structural rearrangements were observed to develop progressively as the extent of reduction of structural Fe(III) increased preventing reversibility upon reoxidation. For instance, reoxidized iron-rich smectites that were initially treated to reduce 100% of structural Fe(III) displayed OH groups in different amount and chemical environment than those treated to reduce only 20% of structural Fe(III). These observations suggested that reversibility was possible only until a certain extent of reduction, which avoided rearrangements in the structure. Jaisi, Dong, and Morton further investigated this statement using biologically and chemically reduced nontronite NAu-2 [40]. The authors uncovered that when the extent of Fe(III) reduction involved less than approximately 30% of total structural Fe(III), the previous mentioned rearrangements in the structure were accommodated in the solid state and initially NAu-2 was restored upon oxidation of structural Fe(II). However, above this percentage, the rearrangements resulted in the development of a progressive amorphous structure for NAu-2. Besides, the accommodation of Fe(II) atoms in the structure resulted in local instability that culminated in partial dissolution of NAu-2 but limited to 35% when 71% of the structural Fe(III) was reduced to Fe(II). After, the fate of Fe(II) released was investigated considering partitioning between the surface, interlayer region and other phases in solution. The authors concluded that released Fe(II) species were preferentially complexed in the surface in early stages of reductive treatment. After partial or total saturation of complexation sites in the surface, the Fe(II) progressively migrated to ion exchange sites replacing sodium cations in the interlayer region until reaching the cation exchange capacity (CEC) value of reduced NAu-2. After this point, further release of structural Fe(II) resulted in aqueous Fe(II) ions.

Chemtob et al. observed a similar behavior for various synthetic ferrous smectites [124]. The extent of oxidation of structural Fe(II) achieved in the presence of O_2_ or H_2_O_2_ solution was compared along with structural alterations resulting from each reaction. The authors reported total oxidation of structural Fe(III) in H_2_O_2_ solution while only partial oxidation was observed in air-saturated solution. However, the greater extent of oxidation of structural Fe(II) observed for H_2_O_2_ also resulted in the ejection of Fe(III) atoms from the octahedral sheet. The authors attributed this observation to increase of local positive charge in trioctahedral clusters that possibly resulted in instability of these domains and ejection of Fe(II), which developed a secondary phase being identified as nanoparticles of hematite.

As for the mechanism behind electron transfer from solution to the solid, Z. Wang used an interesting approach to uncover the role of basal siloxane plane and edge sites [48]. The authors investigated the mechanism behind the electron transfer from the dye Rhodamine B (RhB) excited in the visible range to structural Fe(III) within the octahedral sites of montmorillonite K10 (MK10) and nontronite NAu-2. The oxidation state of structural iron was monitored based on the decay kinetics of H_2_O_2_ as it was assumed that H_2_O_2_ was decomposed by structural Fe(II) via Fenton chemistry. The author separated the basal surface from the edge site using blocking compounds in order to elucidate the major electron-accepting sites. Upon blocking the edge-sites, the authors observed 70% decrease in the decomposition of H_2_O_2_ by NAu-2/dye while extent of decomposition by MK10 was unaltered in this condition. However, upon blocking the basal sites, the decomposition of H_2_O_2_ decreased 50% for K10/dye, but it was unaltered for NAu-2/dye. Both observations were confirmed when the fluorescence of RhB was investigated in the presence of blocking compounds that prevented electron transfer from excited dye molecules to structural Fe(III) sites. The RhB fluorescence decreased 95% for MK10 in the presence of edge site blocker and 42% for NAu-2 in the presence of basal site blocker compound. Both experiments uncovered that the electron accepting sites depend upon the iron content within the clay mineral structure suggesting that higher amount of iron favors mechanism using edge sites while lower amount of iron favors the basal surface.

Latta et al. further discussed these observations and proposed that the acceptance of electrons through the basal plane is favored in poor-iron smectites, such as montmorillonite (SWy-2), because availability of edge Fe(III) and adjacent Fe(III) atoms as clusters is limited throughout the structure in this class of clay minerals [46]. The authors based their study on the oxidation of sorbed Fe(II) on the edge sites. Previously, Schaefer, Gorski, and Scherer also gathered evidence of electron hoping from aqueous 57Fe(II) sorbed in the surface to structural Fe(III) within the octahedral sheet of natural nontronite NAu-2 under anoxic conditions using isotopic Mössbauer spectroscopy for 57Fe [39]. The authors established that data from 57Fe Mossbauer spectroscopy corroborated data obtained for nontronites chemically reduced with dithionite or biologically reduced with bacteria uncovering that structural Fe(III) was reduced by sorbed Fe(II). Besides, the authors showed that the resulting Fe(III) sorbed in the surface resulted in a secondary mineral containing Fe(III) as previously observed by Chemtob et al. [124]. At this time, the authors identified this mineral as being lepidocrocite based on the XRD pattern of NAu-2 following its exposure to Fe(II) in solution.

However, the presence of aqueous Fe(II) is only achieved under anoxic conditions, which may be relevant for groundwater and soil treatment but not for industrial wastewater treatment. Thus, this species should not be considered as possible electron transfer mediators in the Fenton process. Besides, the Fe(II) sorbed in the edges may act as a catalyst rather than an electron transfer mediator in the presence of H_2_O_2_. Still, the studies here reported were considered to enlighten the mechanism regarding electron transfer to and from the lattice of clay minerals that merit further investigation under oxic conditions.

### 4.3. The Catalytic Activity of Structural Fe(II) towards H_2_O_2_ and O_2_

In the literature, the studies involving electrochemistry to reduce Fe(III) within the clay mineral structure are rare and for the most part dedicated to uncovering the electro-activity of resulting Fe(II) sites as mentioned previously [30,44,51]. Besides, to our knowledge, clay-modified electrodes activated with an electron transfer mediator have not yet been explored in the heterogeneous electro-Fenton process. In this approach, the removal of organic contaminants from wastewater would be achieved using •OH radicals generated from structural Fe(II) resulting from electromediated reduction of structural Fe(III). However, several publications have employed photochemistry to reduce structural Fe(III) to Fe(II) that further reacted with O_2_ or H_2_O_2_ to generate •OH radicals. The mechanism behind electron transfer from electron mediator to structural Fe(III) is similar between the electrochemical and photochemical approach and then this latter will be reported in this topic in order to illustrate the efficiency of activated iron-containing lamellar materials to generate •OH radicals and thus to degrade organic contaminants. Most interestingly, the electron transfer mediators in the photochemical approach are the own organic molecules to be degraded in the system that avoid the addition of secondary pollutants. Yet, further studies should be dedicated to elucidating the operational conditions that would allow iron-containing lamellar materials to be reduced in the electrochemical cell in order to subsequently contribute to activation of H_2_O_2_ and thus generation of •OH radicals.

#### 4.3.1. H_2_O_2_

Song et al. evaluated the performance of a natural iron-containing clay mineral, montmorillonite K10 (MK10), in the decomposition of H_2_O_2_ into •OH radicals under visible light [103]. The MK10 was able to decompose 57% of H_2_O_2_ in solution under visible light after 8 h. Under the same conditions, the synthetic hectorite (Laponite) containing only 0.01% of iron resulted in negligible decomposition of H_2_O_2_. This observation confirmed the essential role of structural Fe(III) on the catalytic performance of MK10. However, the MK10 displayed Fe(III) simultaneously in the lattice and as an impurity in the form of iron oxides. Then, the authors pretreated MK10 with sodium dithionite in order to eliminate iron oxides within the structure and thus distinguish reactivity between both iron-containing minerals. After treatment of MK10, C-MK10 was able to decompose 20% of H_2_O_2_ in solution under visible light after 8 h uncovering that 37% of the previous performance was related to impurities in the MK10. However, the addition of *N*,*N*-dimethylaniline (DMA), Rhodamine B (RhB) or Malachite green (MG) enhanced the decomposition of H_2_O_2_ by C-MK10 uncovering photomediated reduction of Fe(III) as key for the performance of C-MK10. The catalytic activity of C-MK10 was demonstrated upon comparison of the previous system with another one only containing N,N-dimethylaniline (DMA), Rhodamine B (RhB) or Malachite green (MG) in which decomposition of H_2_O_2_ was considerably slower than in the presence of C-MK10. The combination of C-MK10 with RhB resulted in decomposition of 94% of H_2_O_2_ in solution under irradiation after 4 h. The authors showed that the fluorescence of RhB was quenched in the presence of C-MK10 and this observation was interpreted as indirect evidence of an electron transfer from RhB to Fe(III) sites in the octahedral sheet of C-MK10. Besides, the authors evaluated the contribution of leached iron to the observed performance of C-MK10. The iron ions in solution were responsible for the decomposition of 13% of H_2_O_2_ in the C-MK10/H_2_O_2_ system. As for the mechanism, the authors proposed that the photoreactive species adsorbed on the surface were excited under UV-vis light and donated an electron to reduce the structural Fe(III) to Fe(II) that further catalyzed the decomposition of H_2_O_2_ to •OH radicals that are readily consumed by the organic species adsorbed in the surface.

After, the same group of researchers investigated reactivity of Fe(III) towards H_2_O_2_ when incorporated into the interlayer space of purified C-MK10 (Fe(III)-C-MK10) and the performance of this system was then compared with C-MK10 in the presence of Malachite green (MG) under visible light [101]. In the case of C-MK10, the authors reported observations similar to their previous study describing degradation of MG in the system C-MK10/H_2_O_2_ only under visible light with complete discoloration after 120 min and TOC decrease of 42% for MG at pH 5. Under the same conditions, a TOC decrease of 50% was observed for MG in the Fe(III)-C-MK10/H_2_O_2_ system under visible light, revealing that the Fe(II) in the interlayer region performed better than structural Fe(II) in the octahedral sheet. Yet, the opposite was previously reported in the absence of visible light [125]. Besides, the authors reported minimal leaching for Fe(III)-C-MK10 and no leaching for C-MK10 validating that Fe(III) atoms within the octahedral sheet of MK10 resulted into a more stable heterogeneous catalyst. This observation was validated upon reuse of C-MK10 in 14 cycles without significant loss of catalytic activity towards H_2_O_2_. As for the mechanism, the authors proposed that structural Fe(III) is not able to form a photoactive complex with H_2_O_2_ as the Fe(III) ions in the interlayer region. This complex is readily reduced to generate HO_2_• radicals and Fe(II) ions further react with H_2_O_2_ to generate •OH radicals. As for the structural Fe(III), it must first undergo photoactivation with excited MG molecules in order to reduce to structural Fe(II) and then decompose H_2_O_2_ into •OH radicals. Therefore, the overall amount of radicals was possibly inferior to the one observed for Fe(III) ions in the interlayer space. However, this mechanism does not agree with data from electron paramagnetic resonance (EPR) using 5,5-dimethyl-1-pyrroline-*N*-oxide (DMPO) as a trapping agent. In the reported data, no signal was observed for C-MK10/H_2_O_2_ system and no other radical species apart from •OH radicals were detected for the Fe(III)-C-MK10/H_2_O_2_ system. Besides, the addition of isopropanol to act as a •OH scavenger did not impact the degradation of MG in the C-MK10/H_2_O_2_ system, which was reported to rely exclusively in •OH radicals. It has to be noted that this observation possibly indicates the contribution of direct oxidation of MG by H_2_O_2_. Regardless, this study encouraged the use of other clay minerals, such as nontronite, based on the premise that an increase of structural Fe(III) sites would enhance the performance of this system.

In this context, R. Liu et al. employed the dye Rhodamine B (RhB) as a photoreactive specie to evaluate the performance of a nontronite (NAU) prepared using a hydrothermal method in the removal of this dye under visible light [126]. In their study, the authors expected to excite the dye molecules with irradiation of light in the visible range in order to promote redox cycling of structural Fe(III) to Fe(II). Then, the RhB was allowed to reach equilibrium of adsorption/desorption 1h before irradiation with visible light. Under dark conditions, the removal of RhB was negligible. Under visible light, the removal of dye in the RhB/H_2_O_2_ system was significantly slower than in the RhB/H_2_O_2_/NAU in which RhB was promptly and completely removed from aerated and deaerated solutions. These observations suggested that •OH radicals were catalytically generated by NAU. After reusing it in six cycles, the authors reported virtually the same catalytic activity and claimed that concentration of free iron ions in solution was undetectable using atomic absorption Spectroscopy. Besides, the preservation of physical and chemical properties of NAU was confirmed upon comparison of their infrared spectra before and after reaction with dye. However, previous studies showed that the structure of nontronites was prone to dissolution when pH was below 5. The redox cycling of structural Fe(III)/Fe(II) resulted in irreversible changes in the structure that were seen in the shift of absorption frequency related to typical vibration modes of the structure [59,127]. Therefore, the observations reported by Liu et al. rather indicate that the redox-cycling of structural Fe(III)/Fe(II) promoted by excited molecules involved a relatively small fraction of total Fe(III) in the structure. As for the mechanism, the authors interpreted that structural Fe(III)/Fe(II) as an electron-shuttle capable of transferring electrons from excited molecules to H_2_O_2_.

#### 4.3.2. O_2_

The previous topic showed that structural Fe(II) is capable of decomposing H_2_O_2_ into •OH radicals. However, such catalytic sites are also capable of reducing O_2_ to H_2_O_2_. Several publications reported that structural Fe(II) generates •OH radical under dark and neutral conditions simply with oxygenation of the surface of Fe(II)-containing minerals, which are common in the subsurface environment. The resulting •OH radicals have been reported to contribute to the oxidation of contaminants in natural sediments (Tong et al., 2016; Liu et al., 2017; Zhou et al., 2019). Then, the studies related to this research topic can also be considered of interest in the context of the heterogeneous electro-Fenton process.

**Figure 9:**
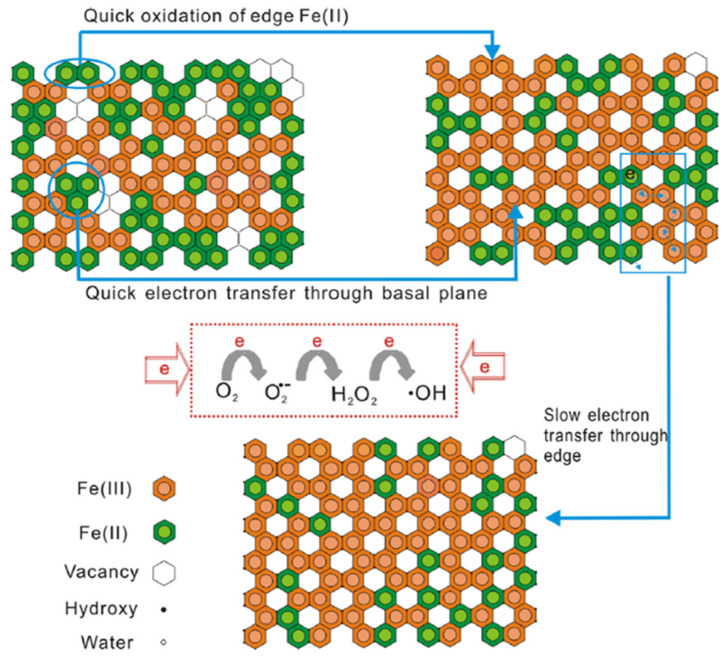
Scheme of the mechanism behind the electron transfer from tri-octahedral or di-octahedral to O_2_ for generation of •OH radicals. Reprinted from [123] with permission from Elsevier (License Number 5172370745843).

The study of Yuan et al. was also able to enlighten the mechanism of electron transfer from Fe(II) within the lattice to O_2_ and then the generation of •OH radicals at neutral conditions based on the identification of structural rearrangements [123]. As previously mentioned, the nontronite NAu-2 was treated with sodium dithionite, which reduced 54.5% of total structural Fe(III) to Fe(II). Upon aeration, the tri-octahedral domains containing Fe(II)-Fe(II)-Fe(II) and edge Fe(II) were oxidized within 15 min while the resulting di-octahedral domains Fe(II)-Fe(II) and others such as Fe(II)-Al(III) and Fe(II)-Fe(III) were slowly oxidized in the interior of the structure. The authors related this observation to the pathway of electrons from the lattice to O_2_. The tri-octahedral domains are more reactive because the electrons supplied from the interior of the structure are possibly ejected through the basal siloxane plane to O_2_ while the electrons resulting from di-octahedral domains migrate through Fe(II)-O-Fe(III) linkages until the edges being there ejected to O_2_. In this case, the presence of Fe in the same oxidation state, inert metals or vacancy sites contribute to retard or obstruct electron migration towards the edge. Furthermore, the Fe(II) content determined after oxidation of reduced NAu-2 by XPS was greater than the one determined by acidic dissolution suggesting the accumulation of Fe(II) in the near surface region of NAu-2. In both pathways, the authors proposed that the generation of •OH radicals was based on one-electron reactions with evidence from scavenging experiments involving reagents such as nitro blue tetrazolium (NBT) and catalase that were able to confirm O_2_•^−^ and H_2_O_2_, respectively, as intermediary species. The electrons were transferred from the structural Fe(II) one by one in reactions with O2, O_2_•^−^ and then H_2_O_2_ for generation of •OH radicals as shown in Figure 9. The corresponding reactions are given in Equations (18)–(20). The authors omitted the oxidation of Fe(II) in the reactions and those were previously proposed here in Equations (6)–(8) highlighting Fenton’s reaction in (8). This detailed study was considered of great importance to the field of heterogeneous Fenton process, because it proposes a mechanism for electron transfer from Fe(II)-containing clay minerals to O_2_ and thus encourages further studies related to generation of •OH radicals in the subsurface sediments under dark and oxic conditions and their contribution to the fate of organic pollutants in both soil and water.

O_2_ + e^−^ → O_2_•^−^ (18)

O_2_•^−^ + e^−^ + 2H^+^ → H_2_O_2_ (19)

H_2_O_2_ + e^−^ + H^+^ → HO• + H_2_O (20)

Later, the same group of researchers also considered the possible role of microbes in the generation and regeneration of structural Fe(II) sites upon depletion of O_2_ in the subsurface sediments [45]. The sediments were incubated with microbes under anoxic conditions for microbial reduction of structural Fe(III) to Fe(II). The resulting sediments generated •OH radicals in a concentration similar to the ones reduced with chemical reagents. The authors interpreted this observation as an indication that generation of •OH radicals in natural subsurface sediments are sustained in the case of periodic anoxic and oxic conditions. Besides, the authors evaluated the generation of •OH in natural conditions. Firstly, the injection of oxygenated water into an aquifer at a depth of 23 m followed by the dosage of •OH radicals confirmed their generation under such conditions. Secondly, the oxygenation of a solution prepared with sediments extracted from the subsurface at a depth of 5 m containing As(III) and tetracycline (antibiotic) confirmed that •OH radicals generated upon oxygenation were able to oxidize contaminants followed by the dosage of As(III)/As(V) and tetracycline in solution. The latter observation encouraged a later study of this research group in which (Liu et al., 2017) investigated the oxidation of trichloroethylene (TCE) by •OH radicals generated from oxygenation of reduced nontronite under neutral conditions.

In this subsequent study, the authors uncovered that an aqueous suspension with 2 g/L of reduced NAu-2 in which 50% of total iron content corresponded to Fe(II) was able to oxidize 89.6% of trichloroethylene (TCE) (1 mg/L) after oxygenation for 3 h under neutral conditions (Liu et al., 2017). First, the removal of TCE under anoxic conditions was confirmed to be negligible. After, the possibility of TCE removal due to evaporation or adsorption on NAu-2 was successfully eliminated in preliminary experiments. Then, the authors investigated parameters influencing the generation of •OH radicals and as a consequence the oxidation of TCE, such as the dosage of NAu-2 and the reduction extend of structural Fe(III) within NAu-2. The cumulative concentration of •OH radicals increased proportionally with the dosage of reduced NAu-2 up to 4 g/L. Above this dosage, structural Fe(II) and TCE competed for resulting •OH radicals and only a slight increase in the oxidation of TCE was observed in this conditions. Therefore, 2 g/L was considered as an ideal dosage of reduced NAu-2 in this study. The reduction extend of NAu-2 was also considered ranging from 13 to 50%. The authors concluded that higher reduction extend was more advantageous than higher dosage of reduced NAu-2 for oxidation of TCE. Besides, the competition between TCE and structural Fe(II) for resulting •OH radicals was not significantly dependent on the reduction extend of NAu-2. Later, the author compared the performance of NAu-2 reduced with sodium dithionite to the one reduced with iron-reducing bacteria. The NAu-2 chemically reduced until 25% showed higher percentages of TCE removal that the one biologically reduced until 30%. Nevertheless, the contribution of the latter for the fate of pollutants in natural subsurface environments was considered of great importance. At last, the reuse of NAu-2 at 2 g/L and reduction extent of 25% in three cycles of oxygenation and (re-)reduction resulted in similar percentages of TCE oxidation indicating the stability of this heterogeneous catalyst. As for the mechanism, the authors also proposed that the generation of •OH radicals proceeded as a two-stage process, as previously proposed by them [123]. The majority of TCE was quickly oxidized within 30 min and then slowly oxidized until the maximum removal was reached within 3 h. This observation was interpreted as an additional evidence that edge Fe(II) sites in the lattice were involved in the first stage while interior electrons were transported through Fe(II)-Fe(III) domains to the edge in order to regenerate Fe(II) sites which were involved in the second stage of the oxidation process. This study showed that the oxidation of TCE under oxic conditions were much faster than its reduction under anoxic conditions uncovering the intrinsic potential of subsurface sediments to remove contaminants when disturbed due to natural processes or human activities.

The systematic analysis of the parameters influencing the oxidation of contaminants is essential to the development of novel remediation processes based on Fe(II)-bearing minerals. In this context, Zeng et al. also developed a study involving nontronite NAu-2 but a cyclic ether compound as a model pollutant, 1,4-dioxane (Zeng et al., 2017). In this study, the authors focused on proposing an efficient protocol for in situ remediation of contaminated water associating Fe(II)-containing clay minerals under dark and neutral conditions. In this context, the authors further investigated parameters influencing the generation of •OH radicals and then the oxidation of 1,4-dioxane under environmental conditions, such as mineral type, mineral dosage and composition of buffer solution. Among montmorillonite (SWy-2), rectorite (RAr-1), illite (IMt-2) and nontronite (NAu-2), the minerals from the smectite group, montmorillonite and nontronite, were considered more advantageous to be used as a catalyst due to the possibility of continuous generation of •OH radicals. Moreover, the reactive Fe(II) sites in nontronite were prone to regeneration following chemical or biological treatment for reduction of structural Fe(III) to Fe(II). However, the authors observed a progressive decrease in the efficiency upon reuse when •OH radicals were generated by chemically reduced nontronite. The opposite was observed for biologically reduced nontronite. In addition, the shifting of absorption bands observed in FTIR spectra as well as the aspect of crystals observed with SEM before and after use and reuse indicated that the greater extent of reduction obtained by chemical treatment caused irreversible structural changes. This changes reduced the efficiency of nontronite to generate •OH radicals. Yet, the authors considered that constant regeneration of catalyst was more advantageous than high dosage as a consequence of the competition of structural Fe(II) with 1,4-dioxane for •OH radicals. As for the mechanism, the authors proposed that structural Fe(II) occupying edge sites were primarily involved in the reduction of O_2_ and then in the oxidation of 1,4-dioxane since the presence of phosphate and citrate in solution increased the generation of •OH radicals by nontronite. Phosphate and citrate are chelating agents capable to strongly adsorb on edge sites upon incorporation of the structural hydroxyl group as a ligand. Then, this observation was interpreted as an evidence that the competitive adsorption of phosphate and citrate on the edges sites favored desorption of •OH radicals in solution. Later, the same group of researchers published a subsequent study using •OH radicals generated by reduced nontronite NAu-2 in phosphate buffer under slightly acidic conditions (pH 6.5) in order to decrease the chemical oxygen demand (COD) of complex wastewater resulting from coking (Zhou et al., 2019).

In this study, other parameters of wastewater were considered such as pH and co-occurrence of other organic pollutants (Zhou et al., 2019). The authors evaluated the convenience of a sequential treatment associating the treatment with chemically reduced NAu-2 and pre- or post-treatment with microbes in order to improve COD removal from effluents. As for pH, the COD removal was 49% and 29% in phosphate buffer under pH 6.5 and 8.2, respectively, after treatment with reduced NAu-2 for 15 days. The authors also investigated the composition of coking wastewater before and after the treatment in order to define which contaminants were more susceptible to be degraded by •OH radicals. Before treatment, the coking wastewater contained mostly aromatic protein-like and humic-like compounds being the latter less susceptible to degradation than aromatic protein-like compounds. This observation was attributed to the higher reaction rate between •OH radicals and unsaturated bonds C=C in comparison to saturated bonds C-C. Then, the coupling of both chemical and biological treatment was proposed in order to increase the obtained COD removal. The sequence biological-chemical was more effective than the chemical-biological resulting in a COD removal of 39.3% and 15.8%, respectively after seven days of treatment in each procedure adding up to 14 days. This observation was related to the nonselective oxidation promoted by •OH radicals that depleted biodegradable compounds and possibly resulted in by-products that are not metabolized or even toxic to the microbes involved in the biological treatment. After treatment, the oxidized nontronite was re-reduced and reused in order to investigate stability of such mineral as a heterogeneous catalyst. The COD removal decreased from 59% to 49% for NAu-2 re-reduced two times and remained 49% for NAu-2 re-reduced three times after cycles of 120 h. This slight reduction in efficiency was attributed to irreversible structural changes, such as dehydroxylation, that resulted from chemical reduction of NAu-2, as mentioned previously.

The studies discussed in this topic exposed the recent advances in the use of iron-rich clay minerals as a catalyst in the heterogeneous Fenton process for degradation of pollutants in water. The urgency behind this research field compelled a relatively fast progress from basic to applied research in the literature. However, further studies are needed, mainly on the use of naturally abundant clay minerals as catalysts for in situ remediation of water and soil based on the heterogeneous Fenton process without supplying H_2_O_2_ or light.

## 5. Future Perspectives and Challenges

The composition of industrial wastewater may require the use of specific systems to treat or eliminate permanent pollutants. Due to changes in the regulatory framework and to the operational cost of these systems (thermal or physical), the development of a new low-cost process is necessary. From an operational point of view, the main challenges that companies are facing are the densification of the treatment systems, their control and efficiency, and the reduction of their operating cost (energy, staff, supplies, waste management). This can be achieved if the nonbiodegradable fraction of the wastewater becomes biodegradable. Then the pretreated wastewater can be sent to a conventional sewage treatment plant. As it has been shown in this review, the electro-Fenton process has many advantages as pretreatment for specific industrial wastewaters.

In this frame, the development of novel electrode materials acting simultaneously as a heterogeneous catalyst and electrode is trending as a possible way to decrease implementation and operational costs. Clay minerals appear as a promising candidate for heterogeneous catalyst because Fe(III) atoms are within the octahedral sheet and tend to remain there after reduction and reoxidation throughout the treatment. This behavior favors a heterogeneous mechanism of catalysis, and the reuse of the catalyst in several treatments [18,19]. The coating of the cathode with iron-rich clay minerals combines the convenience of the treatment at circumneutral pH provided by heterogeneous process with the in situ generation of H_2_O_2_ provided by the electro-Fenton process. The most recent studies in this topic have been critically discussed in this review. The novel composite electrodes based on clay minerals referred to as “particle electrodes” were considered solely as heterogeneous catalyst due to the lack of contact of the particles with the current supply. Therefore, to date, few studies have used clay-modified electrodes in the heterogeneous electro-Fenton process. In these few studies, the efficiency of clay-modified electrodes in the removal of organic pollutants was mainly associated with iron ions leached from the structure due to acidic pH conditions. Besides, high current intensities and long electrolysis were required at circumneutral pH, which are not feasible operational conditions at large scale. The poor performance of clay-modified electrodes may be attributed to structural Fe(III) that poorly catalyze the decomposition of H_2_O_2_ into •OH radical. Thus, the reduction of structural Fe(III) to Fe(II) is key for the performance of iron-rich clay minerals as a heterogeneous catalyst [101,102]. Several studies also discussed in this review have shown that electrons are not directly transferred from the electrode to lattice and the electrochemical reduction of structural Fe(III) is not observed in clay-modified electrodes. For this reason, electron transfer mediators should be associated with clay-modified electrodes in order to assure their performance. However, their use has been neglected in the recent studies.

Therefore, the upcoming studies in the field should focus on detailing the (1) electrochemical properties of clay-modified electrodes (e.g., cyclic voltammetry and conductivity measurements), (2) the mechanical and chemical resistance of clay-modified electrodes to long-term use in the heterogeneous electro-Fenton process, (3) the efficiency related to homogeneous and heterogeneous process during electrolysis, (4) the performance at circumneutral pH in order to avoid adjustment of pH, (5) candidates for electron transfer mediators using either photo- or electrochemistry for the reduction of structural Fe(III) in Fe(II), and (6) the feasibility of large-scale production of proposed materials.

The use of clay-modified electrodes in the presence of electron transfer mediators for the removal of organic pollutants by the heterogeneous electro-Fenton process is considered as a promising research topic in the field. Besides, the use of specific anode materials may allow the electromediated oxidation of pollutants and, as a consequence, further enhance the performance of heterogeneous electro-Fenton systems. At last, the coupling of such heterogeneous electro-Fenton processes with a biological treatment is seen as the most cost-effective treatment for mineralization of pollutants in industrial wastewater. The improvement and the adaptation of this system to the industrial scale is still under development, but the last developments have shown how the electro-Fenton process is adaptable, compact and efficient. The first dimensioning of an industrial electro-Fenton process will be necessary to evaluate the economic viability of this system, before considering its deployment on a larger scale.

## Figures and Tables

**Table 1:** Characteristics of nontronite clay minerals.

Name	Origin	Chemical Formula (INTER[TET][OCT]O_20_(OH)_4_)	Layer Charge		CEC (mEq/100 g)	Surface Area (m^2^/g)	References
			TET	OCT			
Garfield	Garfield, Washington (USA)	Na_0.81_[Si_7.22_Al_0.78_][Fe^3+^_3.64_Fe^2+^_0.01_Al_0.32_Mg_0.04_] O_20_(OH)_4_	−0.78	−0.02	105	-	[24,32]
Panamint Valley	Panamint Valley, California (USA)	Na_0.89_[Si_7.57_Al_0.43_][Fe^3+^_2.87_Fe^2+^_0.01_Al_0.65_Mg_0.47_] O_20_(OH)_4_	−0.43	−0.48	155	-	[24,33]
Ferruginous Smectite (SWa-1)	Grant County, Washington, (USA)	Na_0.87_[Si_7.38_Al_0.62_][Fe^3+^_2.67_Fe^2+^_0.01_Al_1.08_Mg_0.23_] O_20_(OH)_4_	−0.62	−0.27	81	-	[24,34]
NG-1	Hohen Hagen, (Germany)	Na_0.70_[Si_7.29_Fe^3+^_0.63_Al_0.08_][Fe^3+^_3.08_Fe^2+^_0.01_Al_0.88_Mg_0.06_] O_20_(OH)_4_	−0.71	+0.02	97	-	[24,35]
NAu-1	South Australia	M^+^_1.0_[Si_7.00_Al_1.00_][Fe^3+^_3.38_Al_0.58_Mg_0.05_] O_20_(OH)_4_	1.0	-	116	85.3	[36,37,38]
NAu-2	South Australia	M^+^_0.97_[Si_7.57_Fe^3+^_0.42_Al_0.01_][Fe^3+^_3.32_Al_0.52_Mg_0.7_] O_20_(OH)_4_	0.43	0.54	70	28.3–33	[36,39,40,41]

**Table 2:** Characteristics of commonly used montmorillonite clay minerals.

Name	Origin	Chemical Formula (INTER[TET][OCT]O_20_(OH)_4_)	Layer Charge		CEC (mEq/100 g)	Surface Area (m^2^/g)	Reference
			TET	OCT			
MK10	Commercial	-	-	-	100	-	[48]
SWy-1	County of Crook, State of Wyoming (USA)	Ca_0.12_Na_0.32_K_0.05_[Si_7.98_Al_0.02_][Al_3.01_Mg_0.54_Fe^3+^_0.41_Mn_0.01_Ti_0.02_] O_20_(OH)_4_	−0.02	−0.59	76	31	[36,49,50]
SWy-2							
STx-1	County of Gonzales, State of Texas (USA)	Ca_0.27_Na_0.04_K_0.01_[Si_8_][Al_2.41_Mg_0.355_Fe^3+^_0.09_Ti_0.03_] O_20_(OH)_4_	0	−0.59	84	83	

## Glossary

UWWTP	Urban wastewater treatment plants
AOPs	Advanced oxidation processes
R	Organic compounds
•OH	Hydroxyl radical
R-OO•	Peroxyl
HO_2_•	Hydroperoxyl
UV-vis	Ultraviolet and visible light
*h*v	UV irradiation
BDD	Boron-doped diamond
TOC	Total organic carbon
T	Tetrahedral sheet
O	Octahedral sheet
M1	Trans-coordinating octahedral sites
M2	Cis-coordinating octahedral sites
CEC	Cation exchange capacity
SWa-1	Ferruginous smectite
NG-1	Nontronite of Hohen Hagen (Germany)
NAu-1	Nontronites of South Australia
NAu-1	
MK10	Commercial montmorillonite
SWy-1	Montmorillonites of the state of Wyoming (USA)
SWy-2	
STx-1	Montmorillonite of the state of Texas (USA)
EF	Electro-Fenton
HEF	Heterogeneous electro-Fenton
MO	Methyl orange
COD	Chemical oxygen demand
ICE	Intensity of current efficiency
Fe^0^	Zero-valent iron
Fe_2_O_3_	Hematite
Fe_3_O_4_	Magnetite
CF	Carbon felt
LDH	Layered double hydroxides
MBR	Membrane bioreactor
DOC	Dissolved organic carbon
HPLC	High-pressure liquid chromatography
EAOPs	Electrochemical advanced oxidation processes
GCE	Glassy carbon electrode
TLH	Talc-like hybrid
APTES	(3-Aminopropyl)triethoxysilane
bpy	2,2′bipyridine
phen	1,10-phenanthroline
MV^+2^	methyviologen
CPE	Carbon paste electrode
XPS	X-ray photoelectron spectroscopy
RhB	Rhodamine B
MG	Malachite green
DMA	*N*,*N*-dimethylaniline
EPR	Electron paramagnetic resonance
DMPO	5,5-dimethyl-1-pyrroline-*N*-oxide
NBT	Nitro blue tetrazolium
TCE	Trichloroethylene
